# Evolution of cooperation and consistent personalities in public goods games

**DOI:** 10.1038/s41598-021-03045-w

**Published:** 2021-12-09

**Authors:** Mohammad Salahshour

**Affiliations:** Max Planck Institute for Mathematics in the Sciences, Inselstrasse 22, 04103 Leipzig, Germany

**Keywords:** Statistical physics, thermodynamics and nonlinear dynamics, Evolutionary theory, Social evolution, Computational models

## Abstract

The evolution of cooperation has remained an important problem in evolutionary theory and social sciences. In this regard, a curious question is why consistent cooperative and defective personalities exist and if they serve a role in the evolution of cooperation? To shed light on these questions, here, I consider a population of individuals who possibly play two consecutive rounds of public goods game, with different enhancement factors. Importantly, individuals have independent strategies in the two rounds. However, their strategy in the first round affects the game they play in the second round. I consider two different scenarios where either only first-round cooperators play a second public goods game, or both first-round cooperators and first-round defectors play a second public goods game, but in different groups. The first scenario can be considered a reward dilemma, and the second can be considered an assortative public goods game but with independent strategies of the individuals in the two rounds. Both models show cooperators can survive either in a fixed point or through different periodic orbits. Interestingly, due to the emergence of a correlation between the strategies of the individuals in the two rounds, individuals develop consistent personalities during the evolution. This, in turn, helps cooperation to flourish. These findings shed new light on the evolution of cooperation and show how consistent cooperative and defective personalities can evolve and play a positive role in solving social dilemmas.

## Introduction

As cooperation is costly, an individual is better off not cooperating. This leads to a tragedy of the commons where everyone ends up being worse off than if otherwise, all had cooperated^1^. However, contrary to what a rational argument suggests, empirical evidence shows that tragedies of the commons are not that common in nature^2^. Over the past decades, many efforts have been devoted to understanding how evolution has prevented tragedies of the commons and promoted high levels of cooperation^2–5^. Prisoner’s dilemma, and its extension to *n* players, public goods game (PGG), have been the most common frameworks in many of these studies^3,3–6^. In the latter game, each player in a group of *n* players can decide whether to invest an amount *c* in a public good or not. All the investments are multiplied by an enhancement factor $r<n$ and are divided equally among the players. Defectors, refraining from investment, receive the highest payoff and are expected to dominate the population. This expectation contradicts observation^2,3^. Past researches have revealed different mechanisms through which this puzzle can be solved. For instance, when interactions are repeated, cooperation can evolve due to the threat of retaliation of a defective act in future rounds^3,7,8^. However, in many cases, interactions are not repeated. A similar mechanism, indirect reciprocity^8–11^, based on which an individual’s reputation determines others’ behavior towards the individual can be at work to promote cooperation in non-repeated interactions. Similarly, punishment of defectors^12–19^ or rewarding cooperators^20–22^ can promote social behavior. Cooperation can also evolve when interactions are not obligatory^23,24^, or when individuals have a choice between different institutions^25,26^. Furthermore, it is shown that the very existence of population structure can promote cooperation due to the assortativity of interactions in structured populations^5,27^. Other studies have shown assortativity^28^, for instance resulting from kin selection, group selection^4^ or tag-based mechanisms^29^, social diversity^30,31^, heterogeneity^32–34^, conformity^35,36^, costly signaling^37, 38^, moral norms^11,39^, and coevolution of cooperation and language^40^, to mention a few, can play a positive role in the evolution of cooperation.

Despite the valuable insights reached on the subject, open questions regarding the mechanisms and conditions under which cooperative behavior is expected to flourish, remain to be addressed. A curious question in this regard is the existence of consistent cooperative and defective personalities. For instance, public goods experiments have shown that while about half of the people are conditional cooperators who are willing to cooperate provided their group-mates cooperate, others tend to free-ride on others’ contributions^41–44^. Similar observations regarding consistent personality differences in humans and animals, in different contexts, have been made^45^. These observations raise the question of how such consistent cooperative and defective personality differences evolve, and if this can play a constructive role in the evolution of cooperation?

To gain a better insight into this question, here, I consider a context where individuals possibly play two consecutive PGGs. Individuals have independent strategies in the two PGGs. However, their strategy in the first PGG determines the PGG they enter for the second round. This feature of the model has similarities with stochastic games^46^, where individuals’ strategy can affect the game they play in future rounds. However, while in stochastic games, the future interaction occurs in the same context for all the individuals^46^, here, I consider a situation where individuals may have different future interactions based on their strategies. I consider two different scenarios. In the first scenario, only first-round cooperators play a second PGG. In the second scenario, both first-round cooperators and first-round defectors are offered the chance to play a second PGG, but in different groups. In the first scenario, playing a second public good can be considered as a potential reward for cooperative behavior. This scenario is motivated by the observations that in many contexts, cooperation serves as a signal of merit^9,37,38,47^ or offers a high social status^48–50^ which can increase others’ willingness to interact with a cooperator. This can increase an individual’s chance of having future interaction, which I model by allowing a cooperator to enter a second PGG. However, in contrast to a certain reward for a cooperative act, the outcome of the second PGG can be positive or negative, depending on the groups’ ability to solve the second public goods dilemma, which I call a prosocial reward dilemma. Then, the question arises whether the community can solve a reward dilemma and if this can promote cooperation in the first PGG in the first place?

In the second scenario, I consider an assortative context where not only first-round cooperators, but both first-round cooperators and first-round defectors enter a second PGG, but in different groups. This scenario is motivated by many pieces of evidence of assortative behavior^51,52^, such as breaking or forming ties^53,54^, according to which individuals are more likely to interact with similar individuals, and can be considered as a context where both cooperative and defective behavior are rewarded, by, respectively, a prosocial and an antisocial reward dilemma. Importantly, in the model introduced here, the strategies of the individuals are independent in the two rounds. Thus, while it is noted that associativity can promote cooperation when individuals have the same strategy in different interactions - a fact which in a sense underlies many mechanisms for the evolution of cooperation such as network reciprocity, tag based mechanisms, group selection and kin selection - it is not clear whether assortativity of the interactions can play a positive role when individuals have a priori independent strategies in different interactions.

The analysis of the models show that cooperation evolves in both the presence of a reward dilemma and in the presence of assortative interaction. As the comparison of the two scenarios reveals, offering defectors the chance to play a second PGG, that is, an antisocial reward dilemma, can have a surprisingly positive impact on the evolution of cooperation. Interestingly, in an assortative context, in the course of evolution, individuals tend to develop consistent strategies in the two consecutive games. By increasing the likelihood that the benefit of a cooperative act is reaped by fellow cooperators, such a personality consistency, in turn, facilitates the evolution of cooperation. These findings shed new light on the evolution of consistent personalities^45,55–59^, and shows how in an attempt to solve social dilemmas, evolution may have given rise to the evolution of consistent cooperative and defective personalities.

## The model

In our model, in a well-mixed population of *N* individuals, groups of size *g* are randomly formed to play a PGG with enhancement factor $r_1$, possibly followed by a second PGG with enhancement factor $r_2$. That is, at each time step, the whole population is divided into *N*/*g* randomly formed groups. Strategies of the individuals in the second PGG are independent of the first PGG. Thus, there are four possible strategies: cooperation in both games ($C_1C_2$), cooperation in the first game and defection in the second one ($C_1D_2$), defection in the first and cooperation in the second game ($D_1C_2$), and finally, defection in both games ($D_1D_2$). I consider two different scenarios. In the first scenario, called a (prosocial) reward dilemma, while defection entails no more round of PGG, cooperation in the first game leads to the entrance to a second PGG. This scenario is consistent with a situation where cooperation serves as a signal of merit^9,37,38,47^ or offers a high social status^48–50^ which can increase others’ willingness to interact with a cooperator, and thus, the individual is permitted to enter an elite PGG. In the second scenario, called the assortative public goods game, all the individuals in the group proceed to play a second PGG. However, motivated by many pieces of evidence of assortative behavior^51,52^, such as breaking or forming ties^53,54^, I assume individuals are sorted based on their strategies in the first round, such that all the individuals who cooperate in the first round form a subgroup to play a PGG (which is called the cooperative or prosocial PGG), and all those who defect in the first round form a different subgroup to play their second PGG (which is called the defective or anti-social PGG). In this way, the corresponding PGG can be considered an assortative PGG, in which individuals are sorted based on their strategy in the first round.

Individuals gather payoff according to the outcome of the games. Besides, I assume individuals receive a base payoff $\pi _0$ from other activities not related to the PGG. After playing the games, individuals reproduce with a probability proportional to their payoff, such that the population size remains constant. That is, the whole population is updated synchronously such that each individual in the next generation is offspring to an individual *i* in the past generation with a probability proportional to its payoff $\pi _i$. Offspring inherit the strategy of their parent. However, mutations can occur. Mutations in the strategy of the individuals in each round ($s_1$ and $s_2$, where $s_i$ can be *C* or *D*) occur independently, and each with probability $\nu$. When a mutation occurs, the corresponding variable’s value switches to its opposite value (*C* to *D* and vice versa).

**Figure 1:**
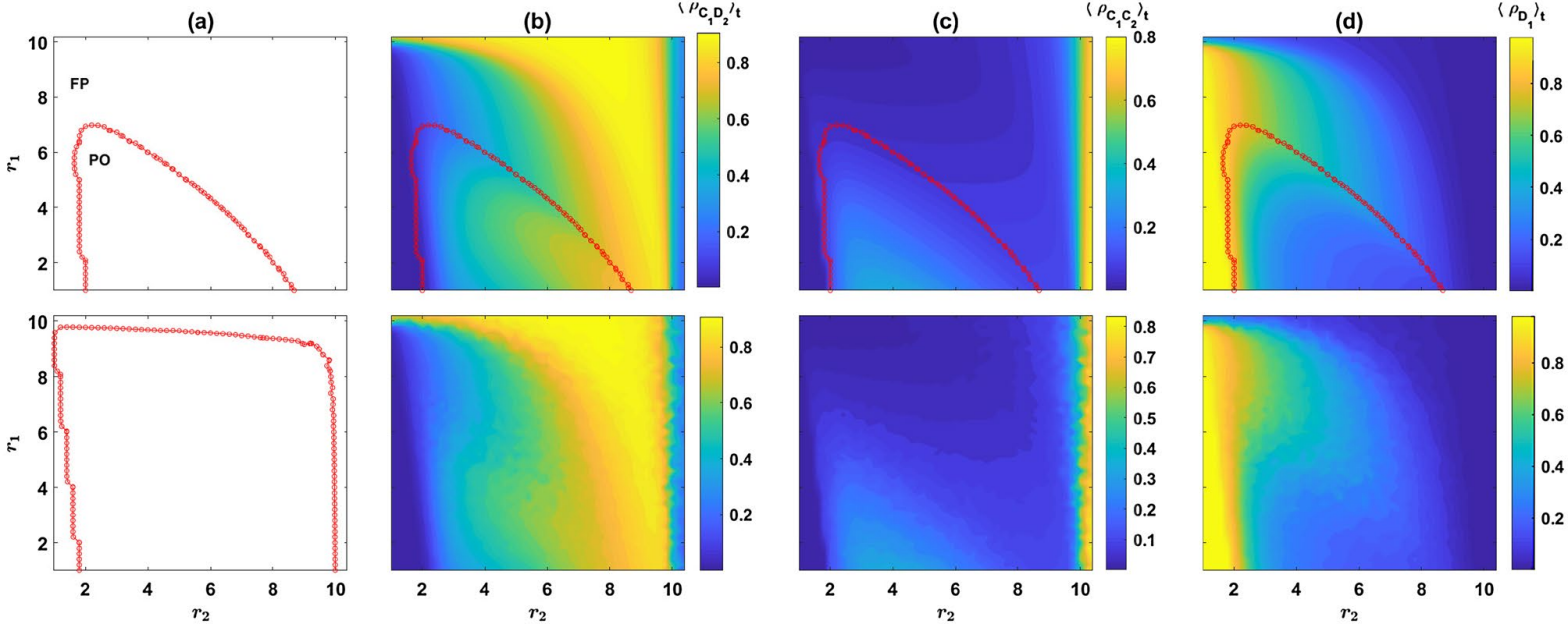
A reward dilemma solves the social dilemma. (**a**) The phase diagram of the model derived from solutions of the replicator dynamics, for two different mutation rates (top $\nu =10^{-3}$ and bottom $\nu =10^{-5}$). The dynamics settle in a periodic orbit or a fixed point depending on the parameters of the model. (**b**)–(**d**) Color plots of, respectively, $\langle \rho _{C_1C_2}\rangle _t$, $\langle \rho _{C_1D_2}\rangle _t$ and $\langle \rho _{D_1}\rangle _t=\langle \rho _{D_1C_1}+\rho _{D_1D_2}\rangle _t$. Top panels result from solutions of the replicator dynamics, and bottom panels result from simulations. Cooperation evolves in the second game (**c**) and is maximized for moderate values of $r_2$. This renders entering the second game an incentive to cooperate in the first game, which promotes cooperation in the first game. Here, $g=10$, $c=1$, and $\pi _0=2$. In (**b**)–(**d**), $\nu =10^{-3}$. Simulations are performed in a population of size $N=5000$. The simulation is performed for $T=3000$ time steps starting from an initial condition with random assignment of strategies, and the time averages are taken over the last 2000 time steps. The replicator dynamics are solved for $T=5000$ time steps starting from homogeneous initial conditions. Time averages are taken over the last 2000 time steps. As the model is mono-stable in the entire phase diagrams, the results are independent of the initial conditions.

## Results

### The first scenario: a reward dilemma solves the social dilemma

As shown in the “Methods” section, both models can be described in terms of the replicator dynamics, which gives an exact description of the model in infinite population limit. Beginning with the reward dilemma model, I note that when $r_2>g$, the second PGG is no longer a dilemma. Cooperation becomes the most rational strategy, which guarantees for the second PGG to yield a positive reward to those who enter it by cooperating in the first PGG. As it is known^20–22^, and as our model confirms, such a certain reward promotes cooperation. The situation becomes more interesting when $r_2<g$. In this case, the second PGG can even yield a negative outcome. For a promise of having future interaction to promote cooperation, the community needs to solve a second dilemma. As I show below, such coupled dilemmas can be solved through evolution.

I begin by plotting the phase diagram of the model for two different mutation rates, $\nu =10^{-3}$ (top), and $\nu =10^{-5}$ (bottom), in Fig. 1a. Here and in the following, $g=10$, $c=1$, $\pi _0=2$. For too large values of $r_1$, the dynamics settle in a fixed point (denoted by FP in the figures). On the other hand, for smaller values of $r_1$, cyclic behavior occurs for intermediate values of $r_2$ (indicated by PO in the figures). For both too large and too small $r_2$, a transition to a phase where the dynamics settle in a fixed point is observed. Comparison of the phase diagram for two different mutation rates shows that lower mutation rates increase the size of the region where the dynamics settle in a periodic orbit.

To see how the cooperation changes with $r_1$ and $r_2$, in Fig. 1b–d, I plot, respectively, the time average of $\rho _{C_1C_2}$, $\rho _{C_1D_2}$ and $\rho _{D_1}=\rho _{D_1C_2}+\rho _{D_1D_2}$. For small $r_2$, such that the second PGG is not profitable enough to motivate cooperation in the first PGG, the dynamics settle into a defective fixed point, where the majority of the individuals defect in the first game and do not play the second game. Consequently $\rho _{D_1}$ takes a large value approximately equal to $1-\nu$, and both $\rho _{C_1C_2}$ and $\rho _{C_1D_2}$ take small values maintained by mutations. As $r_2$ increases, cooperation in both rounds evolve. To see how this happens, I note that the cost of cooperation in the first round is equal to $c(r_1/g-1)$. As a single mutant $C_1C_2$ receives a payoff of $c(r_2-1)$ from the second round, cooperation evolves when the payoff of a mutant $C_1C_2$ from the second round becomes larger than the cost of cooperation in the first round. That is when $r_2>2-r_1/g$. By increasing $r_2$ beyond this point, $\rho _{C_1C_2}$ rapidly increases. This increases the effective group size in the second round PGG, and thus the expected payoff of second-round defectors, which is an increasing function in $\rho _{C_1C_2}$, increases. To see why this is the case, I note that the payoff of a $C_1D_2$ individual from the second round in a group composed of $n_{C_1C_2}$ $C_1C_2$ group-mates and $n_{C_1D_2}$ $C_1D_2$ group-mates is equal to $cr_2\rho _{C_1C_2}/(1+n_{C_1C_2}+n_{C_1D_2})$, which is larger in groups with a higher number of $C_1C_2$ individuals. The probability of formation of groups with a higher number of $C_1C_2$ individuals, in turn, increases with increasing $\rho _{C_1C_2}$ (see “Methods”), and thus, the expected payoff of a second-round defector increases by increasing $\rho _{C_1C_2}$. Consequently $\rho _{C_1D_2}$ increases by further increasing $r_2$ beyond $2-r_1/g$. While this leads to enhanced cooperation in the first round, it also reduces the frequency of second-round cooperators due to the exploitation by second-round defectors. Consequently, the profitability of the second-round PGG decreases and fewer individuals cooperate in the first round to enter the second round PGG. Thus the density of first-round defectors shows a local maximum at a moderate value of $r_2$ at the transition between the cyclic orbit and partially cooperative fixed point at large $r_2$.

**Figure 2:**
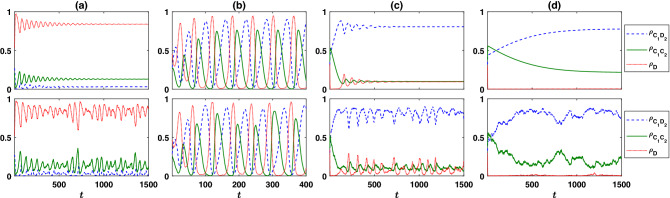
Time evolution of the reward dilemma model. The frequency of different strategies as a function of time is plotted. The top panels represent numerical solutions of the replicator dynamics, and the bottom panel results from a simulation in a population of $N=5000$ individuals. In all the cases, $r_1=1.8$. In (**a**), $r_2=2$, which is slightly larger than the threshold necessary for the evolution of cooperation, $2-r_1/g=1.82$, and thus a small fraction of $C_1C_2$ strategies evolve. In (**b**) $r_2=3.8$, corresponding to the cyclic phase where different strategies cyclically dominate the population. In (**c**) and (**d**), respectively, $r_2=8.8$ and $r_2=9.8$, both corresponding to the cooperative fixed point. Parameter values: $\nu =10^{-3}$, $g=10$, $c=1$, and $\pi _0=2$. The replicator dynamics is solved starting from homogeneous initial conditions, and simulations are performed starting from random assignment of strategies.

The time evolution of the system for different strength of reward dilemma $r_2$ is presented in Fig. 2. Here, the result of the numerical solution of the replicator dynamics (top) and a simulation in a population of size $N=5000$ (bottom) are presented. Here, $g=10$, $\nu =10^{-3}$, $c=1$, $\pi _0=2$, and $r_1=1.8$. In Fig. 2a, $r_2=2$. This is slightly larger than $2-r_1/g=1.82$, and thus $C_1C_2$ strategy survive in the population. For larger values of $r_2$, the fixed point becomes unstable, and the dynamics settle in the cyclic orbit. An example of the cyclic orbit for $r_2=3.8$ is presented in Fig. 2b. In the cyclic phase, when the density of individuals who cooperate in the first round and thus enter the second PGG is small, individuals can reach a high payoff by entering and cooperating in the second PGG. Thus $\rho _{C_1C_2}$ increases. When $\rho _{C_1C_2}$ increases enough, individuals can reach a higher payoff by defecting in the second PGG. At this point, $\rho _{C_1D_2}$ begins to increase, while $\rho _{C_1C_2}$ decreases. As the density of defectors in the second PGG increases, its profitability decreases, and thus individuals have no incentive to cooperate in the first round. Consequently, both $\rho _{D_1D_2}$ and $\rho _{D_1C_2}$, as well as $\rho _D=\rho _{D_1D_2}+\rho _{D_1C_2}$ increase, while other strategies decrease (I note that, since those who defect in the first round do not enter the second game, the two strategies $D_1D_2$ and $D_1C_2$ are degenerate as they lead to the same payoff and are found in the same densities).

The time evolution of the system in the partially cooperative fixed point in large $r_2$ is presented in Fig. 2c,d. In Fig. 2c, $r_2=8.8$. This corresponds to just above the transition line from the periodic solution to the fixed point. Consequently, the replicator dynamics settle in the fixed point after showing transient damped osculations around the fixed point. The simulation results show small fluctuations around the stationary state due to finite-size effects. Comparison of the case of $r_2=3.8$ in Fig. 2b and $r_2=8.8$ in Fig. 2c shows that $\rho _{C_1D_2}$ increases for larger $r_2$ due to the higher profitability of the second-round PGG, which motivates more individuals to cooperate in the first round to enter this PGG. This, in turn, can have an adverse effect on $\rho _{C_1C_2}$ due to the larger effective group size of the second round *PGG*. For larger $r_2$, as in Fig. 2d where $r_2=9.8$, the dynamics settles in the fixed point without showing damped osculations. Furthermore, $\rho _{C_1C_2}$ increases and $\rho _{C_1D_2}$ decrease as $r_2$ approaches *g*.

**Figure 3:**
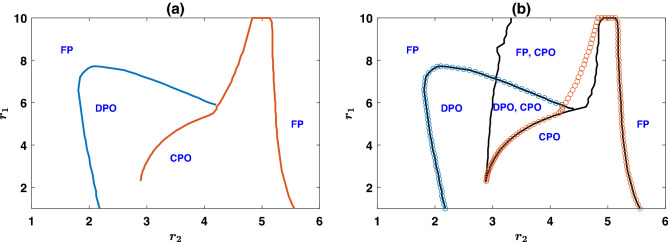
The phase diagram of the assortative public goods game. (**a**) The phase diagram in the $r_1-r_2$ plane. For both small and large values of $r_2$ the model settles into a fixed point (FP). In between, the model shows the cyclic dominance of different strategies. Two different periodic orbits, DPO and CPO, separated by a singular transition exist. (**b**) Different stability regions in the $r_1-r_2$ plane (black lines). The phase boundaries are superimposed (empty circles). The model shows two bistability regions where either both the fixed point and the CPO, or both DPO and CPO are stable. Here, $g=10$, $\nu =10^{-3}$, $c=1$, and $\pi _0=2$.

### The second scenario: the evolution of cooperation and consistent personalities in assortative public goods

As we have seen so far, a reward dilemma solves the social dilemma. An interesting question is whether such a mechanism can be competitive if defectors have the chance of forming a PGG of their own? This brings us to an assortative public goods game where both first-round cooperators and first-round defectors are rewarded by a second round of interaction, but in separate groups.

The phase diagram of the assortative public goods game is presented in Fig. 3a, top panel. For both too small and too large $r_2$, the system settles into a fixed point, and cyclic behavior emerges in between. However, there are two qualitatively different periodic orbits, each stable in some region of the parameter space. For smaller $r_2$, the dynamics settle into a defective periodic orbit (DPO). In this orbit, while cooperation in the cooperative PGG evolves, cooperation does not evolve in the defective PGG. I note that this is the same periodic orbit observed in the reward dilemma model. That such a periodic orbit endures in the second scenario shows its competitive stability. In other words, just as prosocial reward is stable in the presence of an antisocial reward^21^, a prosocial reward dilemma is stable in the presence of an anti-social reward dilemma. On the other hand, for large $r_2$, the dynamics settle into the cooperative periodic orbit (CPO), where cooperation in both cooperative and defective PGGs evolves.

**Figure 4:**
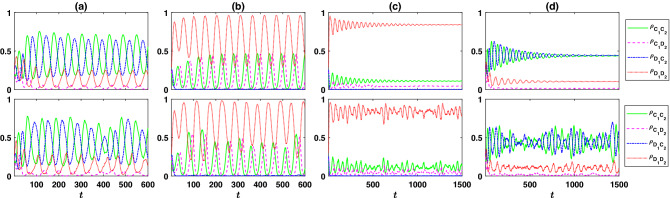
The time evolution of the system in assortative public goods games. (**a**)–(**d**) The frequency of different strategies as a function of time. Top panels show replicator dynamics solutions and the bottom panels result form the simulations in a population of $N=5000$ individuals. The system shows two different periodic orbits. A cooperative periodic orbit, with higher level of cooperation for larger $r_2$, $r_2=4.8$ in (**a**), and a defective periodic orbit, for small $r_2$, $r_2=2.4$ in (**b**). In the fixed point of the dynamics for small $r_2$ but larger than $2-r_1/g$, as in (**c**) where $r_2=2$, only $C_1C_2$ cooperators evolve, and for large $r_2$, $r_2=5.6$ in (**d**), both $C_1C_2$ and $D_1C_2$ evolve. Here, $g=10$, $\nu =10^{-3}$, $c=1$, $\pi _0=2$, and $r_1=2.8$. The replicator dynamics is solved starting from homogeneous initial conditions and simulations are performed starting from random assignment of strategies.

Interestingly, for small fixed $r_1$, by increasing $r_2$ the system shows a cross-over from the DPO to CPO without passing any singularity. However, for large $r_1$, starting from a uniform initial condition, in which the initial densities of all the strategies are equal, as $r_2$ increases, the equilibrium state of the system changes singularly in a certain value of $r_2$. This indicates the transition between DPO and CPO resembles a discontinuous transition for larger $r_1$. As a discontinuous transition is usually accompanied by bistability^60^, this raises the question of whether the system possesses a bistable region as well? To address this question, I present the boundaries of bistability in Fig. 3b (black lines). The phase boundaries, which result from a homogeneous initial condition, are superimposed in this figure as well. The boundaries of bistability are derived by solving the replicator dynamics starting from different initial conditions and checking for hysteresis (see “Methods”). The system is monostable outside of the bistable region, indicated by black lines. In the monostable regions, the dynamics settle in the same stationary state, starting from all the initial conditions. In the bistable region (inside the black line), two different stable states, indicated in the figure, are possible depending on the initial conditions. I note that, while the cooperative periodic orbit is stable in the bistable region, it has a very small basin of attraction, such that the replicator dynamics does not settle into this orbit starting from most randomly generated initial conditions (see the Supplementary Information, figures SI.4 and SI.5). For this reason, to derive the boundaries of bistability, a hysteresis analysis is used (see “Methods”).

As can be seen in the figure, the transition from a fixed point to the DPO in small $r_2$ does not show any bistability and occurs at the same value for all the initial conditions. Similarly, the transition from CPO to a fixed point in large $r_2$ does not show any bistability. In contrast, the transition to CPO by increasing $r_2$ shows bistability: For medium $r_2$, there is a region of the phase diagram where both CPO and DPO are stable. Similarly, the model shows a bistability region for large $r_1$ where both the fixed point and the cooperative periodic orbit are stable.

In Fig. 4, I present the time evolution of different strategies. An example of different periodic orbits is presented in Fig. 4a (CPO) and Fig. 4b (DPO). Top panels present the result of the replicator dynamics, and the bottom panels present the result of a simulation in a population of size $N=5000$. In Fig. 4a, $r_1=2.8$ and $r_2=4.8$, and in Fig. 4b, $r_1=2.8$ and $r_2=2.4$. For larger $r_2$, as in Fig. 4a, defective and cooperative PGGs perform competitively, and competition between these two maintains cooperation in the system. When $\rho _{C_1D_1}$ ($\rho _{D_1D_2}$) is small, the cooperative (defective) PGG is profitable, and thus, it motivates individuals to cooperate (defect) in the first game in order to enter to this PGG. Consequently, $\rho _{C_1C_2}$ ($\rho _{D_1C_2}$) increases. As $\rho _{C_1C_2}$ ($\rho _{D_1C_2}$) increases enough, the cooperative (defective) PGG becomes vulnerable to defection, due to high frequency of cooperators in this PGG which increase the expected payoff of a second round defector, $\rho _{C_1D_2}$ ($\rho _{D_1D_2}$). At this point, $\rho _{C_1D_1}$ ($\rho _{D_1D_2}$) starts to increase. This in turn decreases the profitability of the cooperative (defective) PGG, and individuals are better off by switching to defection (cooperation) in the first round to enter the defective (cooperative) PGG. Consequently, both $\rho _{C_1C_2}$ and $\rho _{C_1D_2}$ ($\rho _{D_1C_2}$ and $\rho _{D_1D_2}$) decrease, while $\rho _{D_1C_2}$ and $\rho _{D_1D_2}$ ($\rho _{C_1C_2}$ and $\rho _{C_1D_2}$) increase. In this way, competition between cooperative and defective PGGs maintain cooperation in the population. Interestingly, individuals tend to have compatible strategies in the two rounds. That is, those who cooperate in the first round are more likely to cooperate in the second round. This can be seen by noting that on average $\rho _{C_1D_2}$ is much smaller than $\rho _{D_1D_2}$, even though defection in cooperative and defective PGGs leads to, on average, similar payoffs as the density of cooperators in these two games ($\rho _{C_1C_2}$ and $\rho _{D_1C_2}$) are similar.

**Figure 5:**
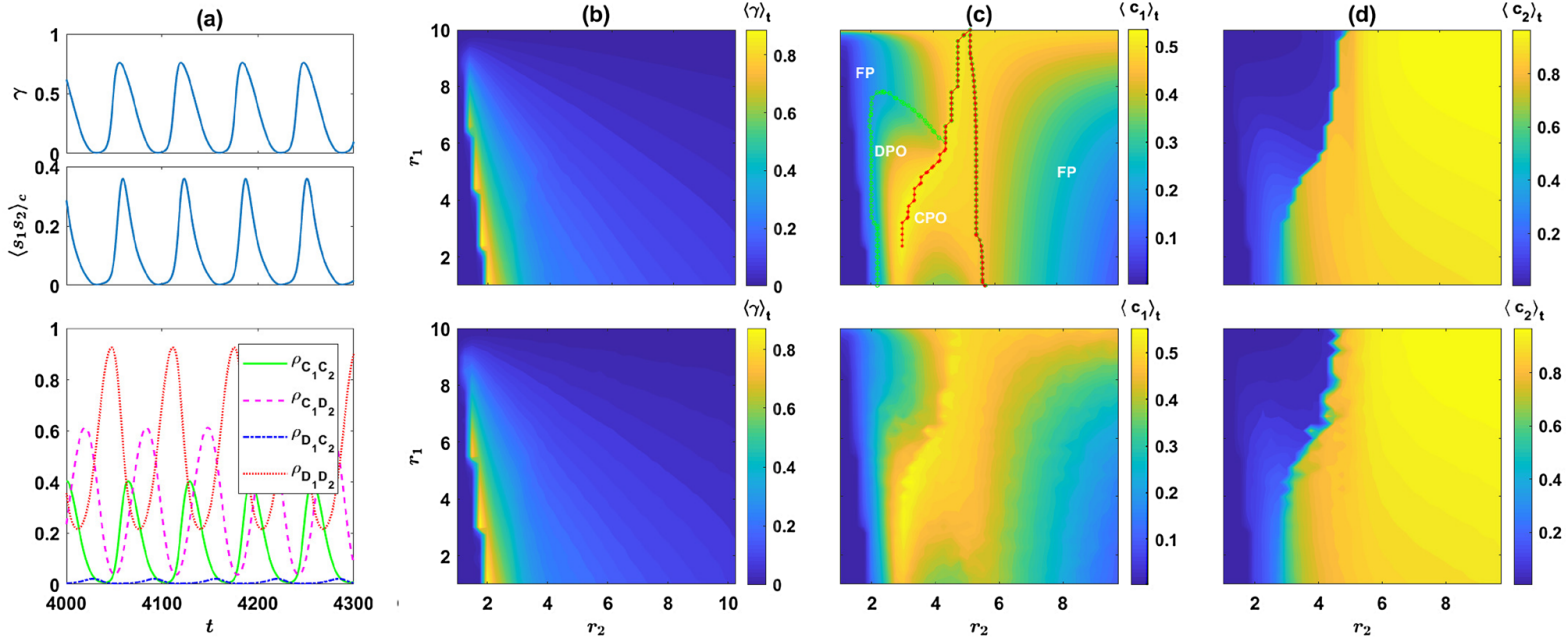
Evolution of cooperation and consistent personalities in assortative public goods game. (**a**) The time series of the personality consistency measure $\gamma$ (top), and the connected correlation function between the strategies of the individuals in the two rounds $\langle s_1s_2\rangle _t$ (middle). For comparison, the densities of different strategies are plotted in the bottom panel. Both measures always remain non-negative. This shows individuals evolve consistent personalities in the two rounds. (**b**) The contour plot of the time average personality measure $\langle \gamma \rangle _t$, in $r_1-r_2$ plane. $\gamma$ remains non-negative in the whole phase diagram, which indicates consistent cooperative and defective personalities evolve. (**c**) The contour plot of the time average cooperation level in the first round $\langle \rho _{C_1}\rangle _t=\langle \rho _{C_1C_2}+\rho _{C_1D_2}\rangle _t$. The phase boundaries are plotted in the top panel as well. Interestingly, the cooperation level in the first round is maximized on the singular transition between the two periodic orbits. (**e**) The contour plot of the time average cooperation level in the second round $\langle \rho _{C_2}\rangle _t=\langle \rho _{C_1C_2}+\rho _{D_1C_2}\rangle _t$. $\langle \rho _{C_2}\rangle _t$ increases with increasing $r_2$. Here, $g=10$, $\nu =10^{-3}$, $c=1$, and $\pi _0=2$. In (**a**) numerical solutions of the replicator dynamics are used. In (**b**)–(**d**), top panels result from numerical solutions of the replicator dynamics, and bottom panels result from simulation on a population of size $N=10{,}000$. The replicator equations are solved for $T=5000$ time steps starting from a homogeneous initial condition, and the time averages are taken over the last 2000 steps. The simulations are performed for $T=4000$ steps starting from a random assignment of strategies, and the averages are taken over the last 3500 steps.

As mentioned before, the situation is different for smaller $r_2$. For smaller $r_2$, as can be seen in Fig. 4b, while cooperation in the cooperative PGG evolves, cooperation in the defective PGG does not evolve. This again hints at the evolution of compatible strategies in the two rounds. Consequently, for smaller $r_2$, while the cooperative PGG can become profitable due to the evolution of fully cooperative $C_1C_2$ strategies and motivates individuals to cooperate in the first game, the defective PGG does not perform competitively and can not attract individuals. As mentioned before, this periodic orbit is the same periodic orbit observed in the reward dilemma model.

The dynamics settle in fixed points for both small and large $r_2$. Example of the dynamics of the system in this regime are presented in Fig. 4c, for small $r_2$, $r_2=2$ and Fig. 4d, for large $r_2$, $r_2=5.6$. As for small $r_2$, cooperation evolves only in the cooperative PGG, the situation is similar to the reward dilemma model: $C_1C_2$ individuals are found in the population (beyond that maintained by mutations) if $r_2>2-r_1/g$. This is what we observe in Fig. 4c. This again hints at the evolution of consistent personalities, as only fist round cooperators cooperate in the second round. The fixed point for large $r_2$ is presented in Fig. 4d. As $r_2$ is chosen just slightly beyond the transition line between cooperative periodic orbit and the fixed point, the dynamics shows damped oscillations around the stationary state before settling in the fixed point. Simulations in a finite population, on the other hand, show that small fluctuations around the stationary state occur in this region. I note that the evolution of consistent personalities can be observed in this regime as well, as the frequency of defectors in the defective PGG is larger than that in the cooperative PGG. That is, first-round defectors are more likely to defect in the second round compared to first-round cooperators.

The fact that individuals’ strategies in the two games tend to be compatible can be studied in more depth. To do this, I consider two measures of consistency of the strategies in the two rounds. As a first measure of compatibility of the strategies of the individuals in the two rounds, I define $\gamma =[P(C_2|C_1)+P(D_2|D_1)-P(D_2|C_1)-P(C_2|D_1)]/2$. Here, $P(s_2|s_1)$ is the conditional probability that an individual has strategy $s_2$ in the second game, given its strategy in the first game $s_1$. $\gamma$ takes a value between one and minus one, and the more positive $\gamma$, the more individuals’ strategies in the two rounds are consistent. As a second measure of personality consistency, I consider the connected correlation function of the strategies of the individuals in the two rounds $\langle s_1 s_2 \rangle _c= \langle s_1s_2\rangle -\langle s_1\rangle \langle s_2\rangle$. To calculate this, I assign $-1$ to the strategy *D*, and $+1$ to the strategy *C*. These are plotted in Fig. 5a (top). For comparison, the density of different strategies is plotted as well (bottom panel). Both personality measures show cyclic behavior and always remain non-negative. Importantly, the latter holds in all the phases. This can be seen in Fig. 5b, where the color plot of the time average of $\gamma$, for numerical solutions of the replicator dynamics (top) and a simulation in a population of size $N=10{,}000$ (bottom) is presented. Interestingly, for fixed $r_2$, personality consistency measures show a maximum close to (but not exactly on) the transition from the fixed point to the defective periodic orbit. This corresponds to $r_2=2-r_1/g$, where the benefit of cooperation in the cooperative PGG starts to become large enough to compensate the cost of cooperation in the first round necessary to enter this PGG. Our results thus show that individuals develop consistent cooperative and defective personalities [see the Supplementary Information for the validity of this result for other parameter values]. This, in turn, plays a positive role in promoting cooperation, as individuals behaving consistently in the two rounds, together with the assortative nature of the public goods, allows first-round cooperators to be more likely to reap the benefit of cooperation in the second round, compared to first-round defectors.

I begin the study of the cooperation by plotting the average cooperation in the first game in Fig. 5c, and the average cooperation in the second game in Fig. 5d. Here, the phase transition lines are indicated in the figure as well. As can be seen, cooperation in the first game is maximized for a moderate value of $r_2$, and it drops for both too small and too large values of $r_2$. Increasing $r_2$ beyond this value has a detrimental effect on cooperation in the first round. This is due to the fact that for larger values of $r_2$, cooperation in the second round increases in both the defective and the cooperative PGGs. This decreases individuals’ incentive to cooperate in the first round to be sorted with fellow cooperators in the second round. On the other hand, for too small $r_2$, any potential benefit from the second round can be too small to promote high cooperation in the first round. Interestingly, the maximum cooperation level is achieved exactly on the transition line between the cooperative and the defective periodic orbits, which coincides with the edge of bistability. This aligns with some arguments that being on the edge of bistability can be beneficial for biological systems^61^.

Finally, the level of cooperation in the second PGG is plotted in Fig. 5d. Here, it can be seen the cooperation increases by increasing $r_2$. On the other hand, as we have seen in Fig. 1, in the reward dilemma model, cooperation in the second game is maximized in an intermediate value of $r_2$. This contrast can be argued to result from competition between the two cooperative and defective PGGs, such that evolution favors the more cooperative one. Consequently, if individuals in one of the two cooperative or defective PGGs start to defect, the other more cooperative one is favored by evolution. This, in turn, shows that a potential reward to defection, by promoting competition, can have a surprisingly positive impact on the evolution of cooperation in an assortative context.

## Discussion

The fact that individuals consistently show cooperative or defective strategies had been noticed in public goods experiments^41–44^, and in many animal populations^45^. It is argued this persistent personality differences can partly explain why cooperation is observed in laboratory experiments and many human and animal societies^41–45^. However, while some theoretical work had shed light on the evolution of some aspects of personality differences, such as the evolution of responsive and unresponsive personalities^57^, risk-averse and risk-taking personalities^55^, or personality differences in leadership^59^, the evolution of cooperative and defective personalities had alluded theoretical understanding. Our findings address this gap by showing how such consistent personality differences can evolve naturally in an evolutionary process when individuals need to work collectively to solve a social dilemma. Importantly, as the analysis of the model shows, the evolution of consistent personalities, in turn, helps solving social dilemmas by increasing the likelihood that the benefit of a cooperative act is reaped by those who behave cooperatively. In this regard, the evolution and maintenance of consistent cooperative and defective personalities can be regarded as an important mechanism at work in promoting cooperation in biological populations.

An interesting question is that why consistent personality differences evolve in an assortative context? The key to the question is that entering the cooperative PGG in the second round is costly, while entering the defective PGG is costless. As it has been shown recently, an entrance cost for a PGG can promote cooperation in a costly PGG, due to the smaller effective size of a costly PGG^26^. For this reason, cooperation is more frequent and defection less frequent in the cooperative, costly PGG, compared to the defective, cost-less PGG. This phenomenon naturally leads to the evolution of consistent cooperative and defective personalities in an assortative context.

Our analysis also reveals new roads to the evolution of cooperation. In this regard, as the analysis of the reward dilemma model shows, a population of self-interested agents can successfully solve a reward dilemma and this, in turn, helps to solve the social dilemma. This mechanism can be at work to promote cooperation in a context where cooperation increases an individual’s chance of having more social interaction, even if actually benefiting from those interactions requires solving another social dilemma. The second scenario model, on the other hand, shows cooperation still evolves when defection is rewarded by a promise of future interaction as well, provided an assortative mechanism is at work. In other words, just as prosocial reward is stable against antisocial reward^21^, a prosocial reward dilemma is competitively stable in promoting cooperation in the presence of an anti-social reward dilemma. In the presence of both prosocial and antisocial reward dilemmas, competition between the prosocial and anti-social public goods maintains cooperation in the system, and moreover, surprisingly increase cooperation in the second round, compared to a case where such competition is lacking. This shows a potential reward to defection, by fostering competition, can have a surprisingly positive impact in promoting cooperation.

## Methods

### Replicator dynamics

The model can be described in terms of replicator-mutation equations^62^, which provide an exact description of the model in infinite population limit. These equations can be written as follows:

1 $$\begin{aligned} \rho _{xy}(t+1)=\sum _{x',y'}\nu _{xy}^{x'y'}\rho _{x'y'}(t)\frac{\pi _{x'y'}(t)}{\sum _{x'',y''}\rho _{x''y''}(t)\pi _{x''y''}(t)}. \end{aligned}$$

Here, *xy* (as well as $x'y'$ and $x''y''$) refer to the strategies of the individuals, such that *x* is the strategy of an individual in the first round, and *y* is its strategy in the second round. *x*, $x'$ etc. can be either cooperation *C* or defection *D*. $\nu _{xy}^{x'y'}$ is the mutation rate from the strategy $x'y'$ to the strategy *xy*. These can be written in terms of mutation rate $\nu$ as follows:

2 $$\begin{aligned} {\left\{ \begin{array}{ll} \nu _{xy}^{x'y'}=1-2\nu +\nu ^2 &{} \quad {\textit{if}}\quad (x=x' \quad {\textit{and}} \quad y=y'),\\ \nu _{xy}^{x'y'}=\nu -\nu ^2 &{}\quad {\textit{if}}\quad (x\ne x' \quad {\textit{and}} \quad y=y')\quad {\textit{or}} \quad (x=x' \quad {\textit{and}} \quad y\ne y'), \\ \nu _{xy}^{x'y'}=\nu ^2&{}\quad {\textit{if}}\quad (x\ne x'\quad {\textit{and}} \quad y \ne y'). \end{array}\right. } \end{aligned}$$

In Eq. (1), $\pi _{x'y'}$ is the expected payoff of an individuals with strategy $x'y'$. In the case of the first scenario, these are given by the following equations:

3 $$\begin{aligned} \pi _{C_1C_2}&=\sum _{n_{D_1C_2}=0}^{g-1-n_{C_1C_2}-n_{C_1D_2}}\sum _{n_{C_1D_2}=0}^{g-1-n_{C_1C_2}}\sum _{n_{C_1C_2}=0}^{g-1}\left[ cr_1\frac{1+n_{C_1}}{g} +cr_2\frac{1+n_{C_1C_2}}{1+n_{C_1}}\right] {\rho _{C_1C_2}}^{n_{C_1C_2}}{\rho _{C_1D_2}}^{n_{C_1D_2}}{\rho _{D_1C_2}}^{n_{D_1C_2}} \\ {}&\quad {\rho _{D_1D_2}}^{g-1-n_{C_1C_2}-n_{C_1D_2}-n_{D_1C_2}}\left( {\begin{array}{c}g-1\\ n_{C_1C_2},n_{C_1D_2},n_{D_1C_2},g-1-n_{C_1C_2}-n_{C_1D_2}-n_{D_1C_2}\end{array}}\right) -2c+\pi _0, \\ \pi _{C_1D_2}&=\sum _{n_{D_1C_2}=0}^{g-1-n_{C_1C_2}-n_{C_1D_2}}\sum _{n_{C_1D_2}=0}^{g-1-n_{C_1C_2}}\sum _{n_{C_1C_2}=0}^{g-1}\left[ cr_1\frac{1+n_{C_1}}{g} +cr_2\frac{n_{C_1C_2}}{1+n_{C_1}}\right] {\rho _{C_1C_2}}^{n_{C_1C_2}}{\rho _{C_1D_2}}^{n_{C_1D_2}}{\rho _{D_1C_2}}^{n_{D_1C_2}} \\ {}&\quad {\rho _{D_1D_2}}^{g-1-n_{C_1C_2}-n_{C_1D_2}-n_{D_1C_2}}\left( {\begin{array}{c}g-1\\ n_{C_1C_2},n_{C_1D_2},n_{D_1C_2},g-1-n_{C_1C_2}-n_{C_1D_2}-n_{D_1C_2}\end{array}}\right) -c+\pi _0, \\ \pi _{D_1C_2}&=\sum _{n_{D_1C_2}=0}^{g-1-n_{C_1C_2}-n_{C_1D_2}}\sum _{n_{C_1D_2}=0}^{g-1-n_{C_1C_2}}\sum _{n_{C_1C_2}=0}^{g-1}\left[ cr_1\frac{n_{C_1}}{g} \right] {\rho _{C_1C_2}}^{n_{C_1C_2}}{\rho _{C_1D_2}}^{n_{C_1D_2}}{\rho _{D_1C_2}}^{n_{D_1C_2}} \\ {}&\quad {\rho _{D_1D_2}}^{g-1-n_{C_1C_2}-n_{C_1D_2}-n_{D_1C_2}}\left( {\begin{array}{c}g-1\\ n_{C_1C_2},n_{C_1D_2},n_{D_1C_2},g-1-n_{C_1C_2}-n_{C_1D_2}-n_{D_1C_2}\end{array}}\right) +\pi _0, \\ \pi _{D_1D_2}&=\sum _{n_{D_1C_2}=0}^{g-1-n_{C_1C_2}-n_{C_1D_2}}\sum _{n_{C_1D_2}=0}^{g-1-n_{C_1C_2}}\sum _{n_{C_1C_2}=0}^{g-1}\left[ cr_1\frac{n_{C_1}}{g} \right] {\rho _{C_1C_2}}^{n_{C_1C_2}}{\rho _{C_1D_2}}^{n_{C_1D_2}}{\rho _{D_1C_2}}^{n_{D_1C_2}} \\ {}&\quad {\rho _{D_1D_2}}^{g-1-n_{C_1C_2}-n_{C_1D_2}-n_{D_1C_2}}\left( {\begin{array}{c}g-1\\ n_{C_1C_2},n_{C_1D_2},n_{D_1C_2},g-1-n_{C_1C_2}-n_{C_1D_2}-n_{D_1C_2}\end{array}}\right) +\pi _0. \end{aligned}$$

Here, we have $n_{C_1}=n_{C_1C_2}+n_{C_1D_2}$. To write these equations, I used the fact that in a group with $n_{C_1C_2}$ individuals with strategy ${C_1C_2}$, and $n_{C_1D_2}$ individuals with strategy ${C_1D_2}$, $r_1\frac{1+n_{C_1}}{g}-c$ and $r_1\frac{n_{C_1}}{g}$ are, respectively, the expected payoff of an individual who cooperates, defects, in the first game. Those who defect in the first game do not gather payoff from the second game. On the other hand, those who cooperate in the first game, obtain a payoff from the second game as well (which can be negative or positive). This is $r_2\frac{1+n_{C_1C_2}}{1+n_{C_1}}-c$ for an individual with strategy $C_1C_2$, and $r_2\frac{n_{C_1C_2}}{1+n_{C_1}}$ for an individual with strategy $C_1D_2$. Finally, ${\rho _{C_1C_2}}^{n_{C_1C_2}}{\rho _{C_1D_2}}^{n_{C_1D_2}}{\rho _{D_1C_2}}^{n_{D_1C_2}}$ $${\rho _{D_1D_2}}^{g-1-n_{C_1C_2}-n_{C_1D_2}-n_{D_1C_2}}\left( {\begin{array}{c}g-1\\ n_{C_1C_2},n_{C_1D_2},n_{D_1D_2},g-1-n_{C_1C_2}-n_{C_1D_2}-n_{D_1C_2}\end{array}}\right)$$, is the probability that a focal individual finds itself in a group with $n_{C_1C_2}$, $n_{C_1D_2}$, $n_{D_1C_2}$, and $n_{D_1D_2}$ individuals with, respectively, strategies $C_1C_2$, $C_1D_2$, $D_1C_2$, and $D_1D_2$. Here, $$\left( {\begin{array}{c}g-1\\ n_{C_1C_2},n_{C_1D_2},n_{D_1D_2},g-1-n_{C_1C_2}-n_{C_1D_2}-n_{D_1C_2}\end{array}}\right)$$ is the multinational coefficients (that is the number of ways that among the $g-1$ group mates of a focal individual, $n_{C_1C_2}$, $n_{C_1D_2}$, $n_{D_1C_2}$, $g-1-n_{C_1C_2}-n_{C_1D_2}-n_{D_1C_2}$ individuals have strategies, respectively, $C_1C_2$, $C_1D_2$, $D_1C_2$, and $D_1D_2$). Summation over all the possible configurations gives the expected payoff of the focal individual with the given strategy from the games. Finally, as all the individuals receive a base payoff $\pi _0$, this is added to the total payoff. Using the expressions in Eq. (3) for the expected payoff of different strategies in Eq. (1), I have a set of four equations which gives an analytical description of the model, in the limit of infinite population size.

In the same way, it is possible to write down equations for the expected payoffs of individuals with different strategies in the second scenario. The difference with the preceding scenario is that, in the second scenario those who defect in the first round proceed to a second PGG as well. Thus, under the same notation and conventions as before, the individuals with strategies $D_1C_2$ and $D_1D_2$, obtain a payoff of, respectively, $r_2\frac{1+n_{D_1C_2}}{1+n_{D_1}}-c$ and $r_2\frac{n_{D_1C_2}}{1+n_{D_1}}$, from their second game. Here, $n_{D_1}=n_{D_1C_1}+n_{D_1D_2}$. Thus, we have for the expected payoffs of different strategies in the second scenario:

4 $$\begin{aligned} \pi _{C_1C_2}&=\sum _{n_{D_1C_2}=0}^{g-1-n_{C_1C_2}-n_{C_1D_2}}\sum _{n_{C_1D_2}=0}^{g-1-n_{C_1C_2}}\sum _{n_{C_1C_2}=0}^{g-1}\left[ cr_1\frac{1+n_{C_1}}{g} +cr_2\frac{1+n_{C_1C_2}}{1+n_{C_1}}\right] {\rho _{C_1C_2}}^{n_{C_1C_2}}{\rho _{C_1D_2}}^{n_{C_1D_2}}{\rho _{D_1C_2}}^{n_{D_1C_2}} \\ {}&\quad {\rho _{D_1D_2}}^{g-1-n_{C_1C_2}-n_{C_1D_2}-n_{D_1C_2}}\left( {\begin{array}{c}g-1\\ n_{C_1C_2},n_{C_1D_2},n_{D_1C_2},g-1-n_{C_1C_2}-n_{C_1D_2}-n_{D_1C_2}\end{array}}\right) -2c+\pi _0, \\ \pi _{C_1D_2}&=\sum _{n_{D_1C_2}=0}^{g-1-n_{C_1C_2}-n_{C_1D_2}}\sum _{n_{C_1D_2}=0}^{g-1-n_{C_1C_2}}\sum _{n_{C_1C_2}=0}^{g-1}\left[ cr_1\frac{1+n_{C_1}}{g} +cr_2\frac{n_{C_1C_2}}{1+n_{C_1}}\right] {\rho _{C_1C_2}}^{n_{C_1C_2}}{\rho _{C_1D_2}}^{n_{C_1D_2}}{\rho _{D_1C_2}}^{n_{D_1C_2}} \\ {}&\quad {\rho _{D_1D_2}}^{g-1-n_{C_1C_2}-n_{C_1D_2}-n_{D_1C_2}}\left( {\begin{array}{c}g-1\\ n_{C_1C_2},n_{C_1D_2},n_{D_1C_2},g-1-n_{C_1C_2}-n_{C_1D_2}-n_{D_1C_2}\end{array}}\right) -c+\pi _0, \\ \pi _{D_1C_2}&=\sum _{n_{D_1C_2}=0}^{g-1-n_{C_1C_2}-n_{C_1D_2}}\sum _{n_{C_1D_2}=0}^{g-1-n_{C_1C_2}}\sum _{n_{C_1C_2}=0}^{g-1}\left[ cr_1\frac{n_{C_1}}{g} +cr_2\frac{1+n_{D_1C_2}}{1+n_{D_1}}\right] {\rho _{C_1C_2}}^{n_{C_1C_2}}{\rho _{C_1D_2}}^{n_{C_1D_2}}{\rho _{D_1C_2}}^{n_{D_1C_2}} \\ {}&\quad {\rho _{D_1D_2}}^{g-1-n_{C_1C_2}-n_{C_1D_2}-n_{D_1C_2}}\left( {\begin{array}{c}g-1\\ n_{C_1C_2},n_{C_1D_2},n_{D_1C_2},g-1-n_{C_1C_2}-n_{C_1D_2}-n_{D_1C_2}\end{array}}\right) -c+\pi _0, \\ \pi _{D_1D_2}&=\sum _{n_{D_1C_2}=0}^{g-1-n_{C_1C_2}-n_{C_1D_2}}\sum _{n_{C_1D_2}=0}^{g-1-n_{C_1C_2}}\sum _{n_{C_1C_2}=0}^{g-1}\left[ cr_1\frac{n_{C_1}}{g} +cr_2\frac{n_{D_1C_2}}{1+n_{D_1}}\right] {\rho _{C_1C_2}}^{n_{C_1C_2}}{\rho _{C_1D_2}}^{n_{C_1D_2}}{\rho _{D_1C_2}}^{n_{D_1C_2}} \\ {}&\quad {\rho _{D_1D_2}}^{g-1-n_{C_1C_2}-n_{C_1D_2}-n_{D_1C_2}}\left( {\begin{array}{c}g-1\\ n_{C_1C_2},n_{C_1D_2},n_{D_1C_2},g-1-n_{C_1C_2}-n_{C_1D_2}-n_{D_1C_2}\end{array}}\right) +\pi _0. \end{aligned}$$

Using these expressions for the expected payoffs of individuals with different strategies in Eq. (1), we have the analytical description of the second scenario model, in the limit of infinite population size.

### The simulations and numerical solutions

Numerical solutions of the replicator dynamics result from numerically solving the replicator dynamics of the models derived in the “Methods” section. Simulations of the models are performed according to the model definition. Unless otherwise stated, both the simulations and numerical solutions of the replicator dynamics are performed with an initial condition in which all the strategies are found in similar frequencies in the population pool. For the solutions of the replicator dynamics, this is assured by setting the initial frequency of all the four strategies equal to 1/4. For simulations, this is assured by a random assignment of the strategies. The phase diagram presented in Fig. 5a is derived by locating the parameter values where a transition between different attractors occurs starting from a homogeneous initial condition. The boundary of bistability in Fig. 5a is derived by examining history dependence and checking for the existence of hysteresis in the evolution of the system. That is, the replicator dynamics are solved starting from parameter values belonging to different phases. Then, the parameter values are changed in small steps, using the stationary state of the preceding steps as the initial condition for the solution of the replicator dynamics in the next step. In this way, the boundary of bistability beyond which a solution becomes unstable is found. See the Supplementary Information for more details.

## Supplementary Information

Supplementary Information.
