# Supplementary material for: Evolution of cooperation and consistent personalities in public goods games
Source: Sci Rep. 2021 Dec 9;11:23708. doi: 10.1038/s41598-021-03045-w (PMC8660839; doi:10.1038/s41598-021-03045-w)
Supplement: Supplementary file 1 — Supplementary Information. [file 41598_2021_3045_MOESM1_ESM.pdf]

Supplementary Information for: Evolution of cooperation and consistent personalities in public goods games

Mohammad Salahshour

SI. 1 Methods

SI. 1.1 Overview of the model

For completeness, here we give an overview of the model. We consider a population of N individuals, who live for two rounds and possibly play a public goods game (PGG) in each round. Individuals have independent strategies in the two rounds. In the beginning of each generation (which we refer to as a time step), groups of size g individuals are formed randomly from the population pool. In the first round, in each group, a PGG with enhancement factor r_1 is played. We consider two different scenarios. In the first scenario, those who cooperate in the first round, proceed to play a second PGG with all those who had cooperated in the first round in their group. Those who defect in the first round can not play a PGG for the second round. In the second scenario, both first round cooperators and first round defectors are able to play a PGG for the second round. However, in the second round, individuals are sorted based on their strategies in the first round. That is, all those who had cooperated in the first round in a group, form a subgroup to play a PGG (which we call the cooperative PGG), and all those who had defected in the first round form another subgroup to play their second PGG (which we call the defective PGG). We assume the second PGG to have an enhancement factor r_2.

Individuals gather payoff according to the outcome of the games. In addition, they receive a base payoff π_0 from other activities not related to the games. After the second round, a selection occurs, during which individuals are selected with a probability proportional to their payoff, such that the population size remains constant. That is each individual in the next generation is offspring to one of the individuals in the past generation with a probability proportional to its payoff. Offspring inherit the strategy of their parent. However, mutation in each strategy can occur with a probability ν, in which case the value of the corresponding strategy is flipped to its opposite value.

SI. 1.2 The simulations and numerical solutions

Analytical solutions result from numerically solving the replicator dynamics of the models, derived in the next section. Simulations of the models are performed according to the model definition. Unless otherwise stated, both simulations and analytical solutions are performed with an initial condition in which all the strategies are found in similar frequencies in the population pool. For the solutions of the replicator dynamics, this is assured by setting the initial frequency of all the four strategies equal to 1/4. For simulations, this is assured by a random assignment of the strategies in the beginning of the simulation. The phase diagram presented in Fig. (2.a) is derived by locating the parameter values where a transition between different attractors occurs starting from a homogeneous initial condition. The boundary of bistabilities is derived by examining history dependence and checking for the existence of hysteresis in the evolution of the system. See section SI. 2.3 for more details.

SI. 1.3 Replicator dynamics

The model can be solved analytically in terms of replicator-mutation equations. These equations can be written as follows:

$$\rho_{xy}(t+1) = \sum_{x',y'} \nu_{xy}^{x'y'} \rho_{x'y'}(t) \frac{\pi_{x'y'}(t)}{\sum_{x'',y''} \rho_{x''y''}(t)\pi_{x''y''}(t)}. \tag{SI.1}$$

Here, xy (as well as $x'y'$ and $x''y''$) refer to the strategies of the individuals, such that x is the strategy of an individual in the first round, and y is its strategy in the second round. x, x' etc. can be either cooperation C or defection D. $\nu_{xy}^{x'y'}$ is the mutation rate from the strategy $x'y'$ to the strategy xy. These can be written in terms of mutation rate ν as follows:

$$\begin{cases} \nu_{xy}^{x'y'} = 1 - 2\nu + \nu^2 & \textit{if} \quad (x = x' \quad \textit{and} \quad y = y'), \\ \nu_{xy}^{x'y'} = \nu - \nu^2 & \textit{if} \quad (x \neq x' \quad \textit{and} \quad y = y') \quad \textit{or} \quad (x = x' \quad \textit{and} \quad y \neq y'), \\ \nu_{xy}^{x'y'} = \nu^2 & \textit{if} \quad (x \neq x' \quad \textit{and} \quad y \neq y'). \end{cases} \tag{SI.2}$$

In eq. (SI.1), $\pi_{x'y'}$ is the expected payoff of an individuals with strategy $x'y'$. In the case of the first scenario, these are given by the following equations:

$$\begin{aligned}
\pi_{C_1C_2} = & \sum_{n_{D_1C_2}=0}^{g-1-n_{C_1C_2}-n_{C_1D_2}} \sum_{n_{C_1D_2}=0}^{g-1-n_{C_1C_2}} \sum_{n_{C_1C_2}=0}^{g-1} \left[cr_1 \frac{1+n_{C_1}}{g} + cr_2 \frac{1+n_{C_1C_2}}{1+n_{C_1}} \right] \rho_{C_1C_2}{}^{n_{C_1C_2}} \rho_{C_1D_2}{}^{n_{C_1D_2}} \rho_{D_1C_2}{}^{n_{D_1C_2}} \\
& \rho_{D_1D_2}{}^{g-1-n_{C_1C_2}-n_{C_1D_2}-n_{D_1C_2}} \binom{g-1}{n_{C_1C_2}, n_{C_1D_2}, n_{D_1C_2}, g-1-n_{C_1C_2}-n_{C_1D_2}-n_{D_1C_2}} - 2c + \pi_0, \\
\pi_{C_1D_2} = & \sum_{n_{D_1C_2}=0}^{g-1-n_{C_1C_2}-n_{C_1D_2}} \sum_{n_{C_1D_2}=0}^{g-1-n_{C_1C_2}} \sum_{n_{C_1C_2}=0}^{g-1} \left[cr_1 \frac{1+n_{C_1}}{g} + cr_2 \frac{n_{C_1C_2}}{1+n_{C_1}} \right] \rho_{C_1C_2}{}^{n_{C_1C_2}} \rho_{C_1D_2}{}^{n_{C_1D_2}} \rho_{D_1C_2}{}^{n_{D_1C_2}} \\
& \rho_{D_1D_2}{}^{g-1-n_{C_1C_2}-n_{C_1D_2}-n_{D_1C_2}} \binom{g-1}{n_{C_1C_2}, n_{C_1D_2}, n_{D_1C_2}, g-1-n_{C_1C_2}-n_{C_1D_2}-n_{D_1C_2}} - c + \pi_0, \\
\pi_{D_1C_2} = & \sum_{n_{D_1C_2}=0}^{g-1-n_{C_1C_2}-n_{C_1D_2}} \sum_{n_{C_1D_2}=0}^{g-1-n_{C_1C_2}} \sum_{n_{C_1C_2}=0}^{g-1} \left[cr_1 \frac{n_{C_1}}{g} \right] \rho_{C_1C_2}{}^{n_{C_1C_2}} \rho_{C_1D_2}{}^{n_{C_1D_2}} \rho_{D_1C_2}{}^{n_{D_1C_2}} \\
& \rho_{D_1D_2}{}^{g-1-n_{C_1C_2}-n_{C_1D_2}-n_{D_1C_2}} \binom{g-1}{n_{C_1C_2}, n_{C_1D_2}, n_{D_1C_2}, g-1-n_{C_1C_2}-n_{C_1D_2}-n_{D_1C_2}} + \pi_0, \\
\pi_{D_1D_2} = & \sum_{n_{D_1C_2}=0}^{g-1-n_{C_1C_2}-n_{C_1D_2}} \sum_{n_{C_1D_2}=0}^{g-1-n_{C_1C_2}} \sum_{n_{C_1C_2}=0}^{g-1} \left[cr_1 \frac{n_{C_1}}{g} \right] \rho_{C_1C_2}{}^{n_{C_1C_2}} \rho_{C_1D_2}{}^{n_{C_1D_2}} \rho_{D_1C_2}{}^{n_{D_1C_2}} \\
& \rho_{D_1D_2}{}^{g-1-n_{C_1C_2}-n_{C_1D_2}-n_{D_1C_2}} \binom{g-1}{n_{C_1C_2}, n_{C_1D_2}, n_{D_1C_2}, g-1-n_{C_1C_2}-n_{C_1D_2}-n_{D_1C_2}} + \pi_0.
\end{aligned} \tag{SI.3}$$

Here, we have $n_{C_1} = n_{C_1C_2} + n_{C_1D_2}$. To write these equations, we used the fact that in a group with $n_{C_1C_2}$ individuals with strategy C_1C_2, and $n_{C_1D_2}$ individuals with strategy C_1D_2, $r_1\frac{1+n_{C_1}}{g} - c$ and $r_1\frac{n_{C_1}}{g}$ are, respectively, the expected payoff of an individual who cooperates, defects, in the first game. Those who defect in the first game do not gather payoff from the second game. On the other hand, those who cooperate in the first game, obtain a payoff from the second game as well (which can be

negative or positive). This is $r_2\frac{1+n_{C_1C_2}}{1+n_{C_1}}-c$ for an individual with strategy C_1C_2, and $r_2\frac{n_{C_1C_2}}{1+n_{C_1}}$ for an individual with strategy C_1D_2. Finally, ${\rho_{C_1C_2}}^{n_{C_1C_2}}{\rho_{C_1D_2}}^{n_{C_1D_2}}{\rho_{D_1C_2}}^{n_{D_1C_2}}{\rho_{D_1D_2}}^{g-1-n_{C_1C_2}-n_{C_1D_2}-n_{D_1C_2}}\binom{g-1}{n_{C_1C_2},n_{C_1D_2},n_{D_1D_2},g-1-n_{C_1C_2}-n_{C_1D_2}-n_{D_1C_2}}$, is the probability that a focal individual finds itself in a group with $n_{C_1C_2}$, $n_{C_1D_2}$, $n_{D_1C_2}$, and $n_{D_1D_2}$ individuals with, respectively, strategies C_1C_2, C_1D_2, D_1C_2, and D_1D_2. Here, $\binom{g-1}{n_{C_1C_2},n_{C_1D_2},n_{D_1D_2},g-1-n_{C_1C_2}-n_{C_1D_2}-n_{D_1C_2}}$ is the multinational coefficient. This is the number of ways that among the $g-1$ group mates of a focal individual, $n_{C_1C_2}$, $n_{C_1D_2}$, $n_{D_1C_2}$, $g-1-n_{C_1C_2}-n_{C_1D_2}-n_{D_1C_2}$ individuals have strategies, respectively, C_1C_2, C_1D_2, D_1C_2, and D_1D_2). Summation over all the possible configurations gives the expected payoff of the focal individual with the given strategy from the games. Finally, as all the individuals receive a base payoff π_0, this is added to the total payoff. Using the expressions in eq. (SI.3) for the expected payoff of different strategies in eq. (SI.1), we have a set of four equations which gives an analytical description of the model, in the limit of infinite population size.

In the same way, it is possible to write down equations for the expected payoffs of individuals with different strategies in the second scenario. The difference with the preceding scenario is that, in the second scenario those who defect in the first round proceed to a second PGG as well. Thus, under the same notation and conventions as before, the individuals with strategies D_1C_2 and D_1D_2, obtain a payoff of, respectively, $r_2\frac{1+n_{D_1C_2}}{1+n_{D_1}}-c$ and $r_2\frac{n_{D_1C_2}}{1+n_{D_1}}$, from their second game. Here, $n_{D_1}=n_{D_1C_1}+n_{D_1D_2}$. Thus, we have for the expected payoffs of different strategies in the second scenario:

$$
\begin{aligned}
\pi_{C_1C_2} = &\sum_{n_{D_1C_2}=0}^{g-1-n_{C_1C_2}-n_{C_1D_2}}\sum_{n_{C_1D_2}=0}^{g-1-n_{C_1C_2}}\sum_{n_{C_1C_2}=0}^{g-1}\left[cr_1\frac{1+n_{C_1}}{g}+cr_2\frac{1+n_{C_1C_2}}{1+n_{C_1}}\right]{\rho_{C_1C_2}}^{n_{C_1C_2}}{\rho_{C_1D_2}}^{n_{C_1D_2}}{\rho_{D_1C_2}}^{n_{D_1C_2}}\\
&{\rho_{D_1D_2}}^{g-1-n_{C_1C_2}-n_{C_1D_2}-n_{D_1C_2}}\binom{g-1}{n_{C_1C_2},n_{C_1D_2},n_{D_1C_2},g-1-n_{C_1C_2}-n_{C_1D_2}-n_{D_1C_2}}-2c+\pi_0,\\
\pi_{C_1D_2} = &\sum_{n_{D_1C_2}=0}^{g-1-n_{C_1C_2}-n_{C_1D_2}}\sum_{n_{C_1D_2}=0}^{g-1-n_{C_1C_2}}\sum_{n_{C_1C_2}=0}^{g-1}\left[cr_1\frac{1+n_{C_1}}{g}+cr_2\frac{n_{C_1C_2}}{1+n_{C_1}}\right]{\rho_{C_1C_2}}^{n_{C_1C_2}}{\rho_{C_1D_2}}^{n_{C_1D_2}}{\rho_{D_1C_2}}^{n_{D_1C_2}}\\
&{\rho_{D_1D_2}}^{g-1-n_{C_1C_2}-n_{C_1D_2}-n_{D_1C_2}}\binom{g-1}{n_{C_1C_2},n_{C_1D_2},n_{D_1C_2},g-1-n_{C_1C_2}-n_{C_1D_2}-n_{D_1C_2}}-c+\pi_0,\\
\pi_{D_1C_2} = &\sum_{n_{D_1C_2}=0}^{g-1-n_{C_1C_2}-n_{C_1D_2}}\sum_{n_{C_1D_2}=0}^{g-1-n_{C_1C_2}}\sum_{n_{C_1C_2}=0}^{g-1}\left[cr_1\frac{n_{C_1}}{g}+cr_2\frac{1+n_{D_1C_2}}{1+n_{D_1}}\right]{\rho_{C_1C_2}}^{n_{C_1C_2}}{\rho_{C_1D_2}}^{n_{C_1D_2}}{\rho_{D_1C_2}}^{n_{D_1C_2}}\\
&{\rho_{D_1D_2}}^{g-1-n_{C_1C_2}-n_{C_1D_2}-n_{D_1C_2}}\binom{g-1}{n_{C_1C_2},n_{C_1D_2},n_{D_1C_2},g-1-n_{C_1C_2}-n_{C_1D_2}-n_{D_1C_2}}-c+\pi_0,\\
\pi_{D_1D_2} = &\sum_{n_{D_1C_2}=0}^{g-1-n_{C_1C_2}-n_{C_1D_2}}\sum_{n_{C_1D_2}=0}^{g-1-n_{C_1C_2}}\sum_{n_{C_1C_2}=0}^{g-1}\left[cr_1\frac{n_{C_1}}{g}+cr_2\frac{n_{D_1C_2}}{1+n_{D_1}}\right]{\rho_{C_1C_2}}^{n_{C_1C_2}}{\rho_{C_1D_2}}^{n_{C_1D_2}}{\rho_{D_1C_2}}^{n_{D_1C_2}}\\
&{\rho_{D_1D_2}}^{g-1-n_{C_1C_2}-n_{C_1D_2}-n_{D_1C_2}}\binom{g-1}{n_{C_1C_2},n_{C_1D_2},n_{D_1C_2},g-1-n_{C_1C_2}-n_{C_1D_2}-n_{D_1C_2}}+\pi_0.
\end{aligned}
\tag{SI.4}
$$

Using these expressions for the expected payoffs of individuals with different strategies in eq. (SI.1), we have the analytical description of the second scenario model, in the limit of infinite population size.

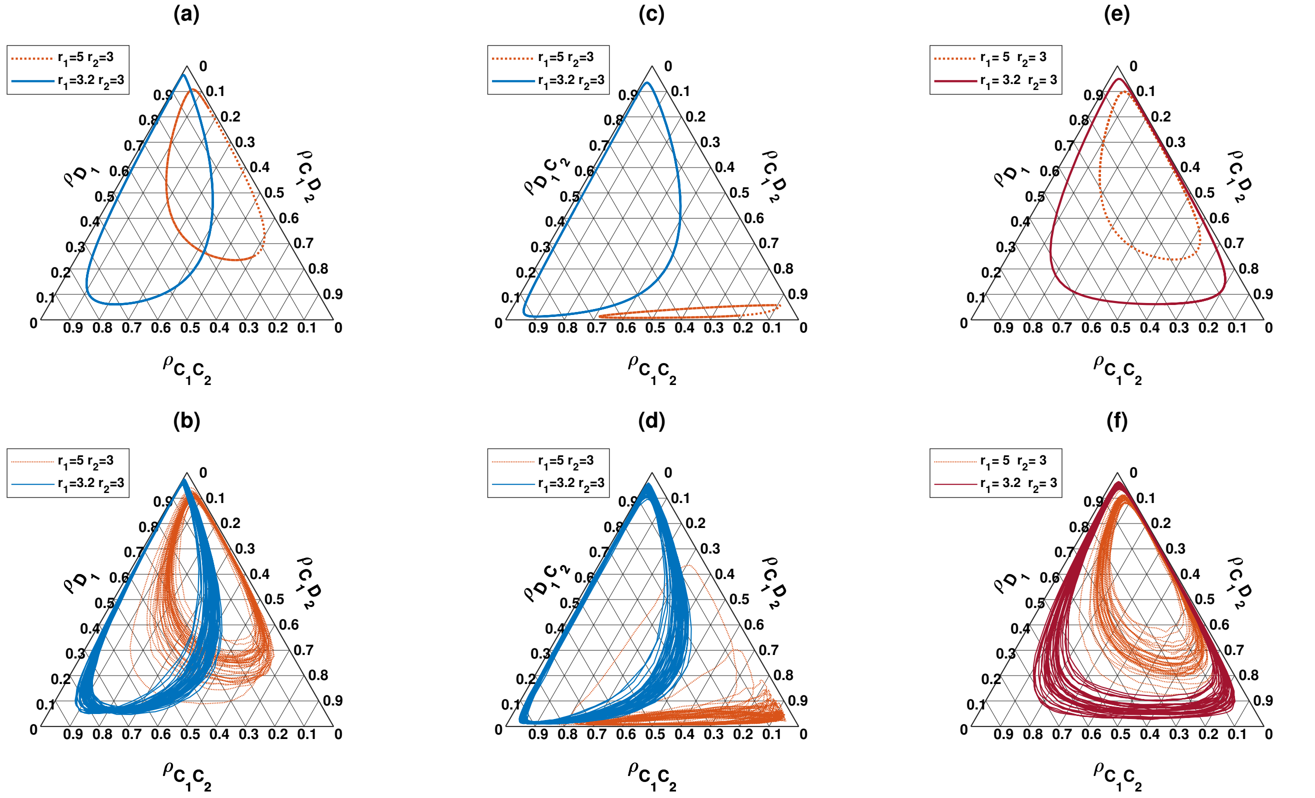

Figure SI.1: Periodic orbits. The periodic orbits in the second scenario model, as it results from the replicator dynamics (a and c), and from a simulation in a population of size $N = 20000$ (b and d). (a) and (b), present the periodic orbits in the $\rho_{D_1} - \rho_{C_1D_2} - \rho_{C_1C_2}$ ternary diagram, and (c) and (d) present the same periodic orbit in $\rho_{D_1C_2} - \rho_{C_1D_2} - \rho_{C_1C_2}$ ternary diagram. The model shows two different periodic orbit, a defective periodic orbit (dotted red) for small r_2 and large r_1, and a cooperative periodic orbit (solid blue) for large r_2 and small r_1. (e) and (f) The periodic orbit in the first Model resulting from numerical solutions of the replicator dynamics (e), and a simulation in a population of size $N = 20000$ (f). These correspond to the defective periodic orbit in the second scenario model. Here, $\nu = 10^{-3}$, $g = 10$, $c = 1$, and $\pi_0 = 2$. In (b), (d), and (f), the simulations is run for $T = 3000$ time steps and the last 2500 time steps are shown.

SI. 2 The periodic orbits and transition between them

SI. 2.1 The periodic orbits in the first and second scenario model

As mentioned in the main text, the second scenario can give rise to two qualitatively different periodic orbits, each stable in some range of the parameter values. Examples of these POs can be observed in the ternary plots in Fig. (SI.1). Here, in each panel, two POs observed for two different values of r_1 and r_2, as specified in the figures are plotted. Here, $g = 10$, $\nu = 10^{-3}$, $c = 1$, and $\pi_0 = 2$. In Fig. (SI.1.a) and Fig. (SI.1.b), the periodic orbits in the $\rho_{D_1} - \rho_{C_1D_2} - \rho_{C_1C_2}$ ternary diagrams are plotted (here, $\rho_{D_1} = \rho_{D_1C_2} + \rho_{D_1D_2}$). The same POs are plotted in the $\rho_{D_1C_2} - \rho_{C_1D_2} - \rho_{C_1C_2}$ ternary diagrams in Fig. (SI.1.c) and Fig. (SI.1.d). In Fig. (SI.1.a) and Fig. (SI.1.c), the analytical solutions are used, and in Fig. (SI.1.b) and Fig. (SI.1.d), a simulation in a population of size $N = 20000$ is used.

For small r_2, in a fixed r_1 (or large r_1 with a fixed r_2) chosen below the the transition line, the

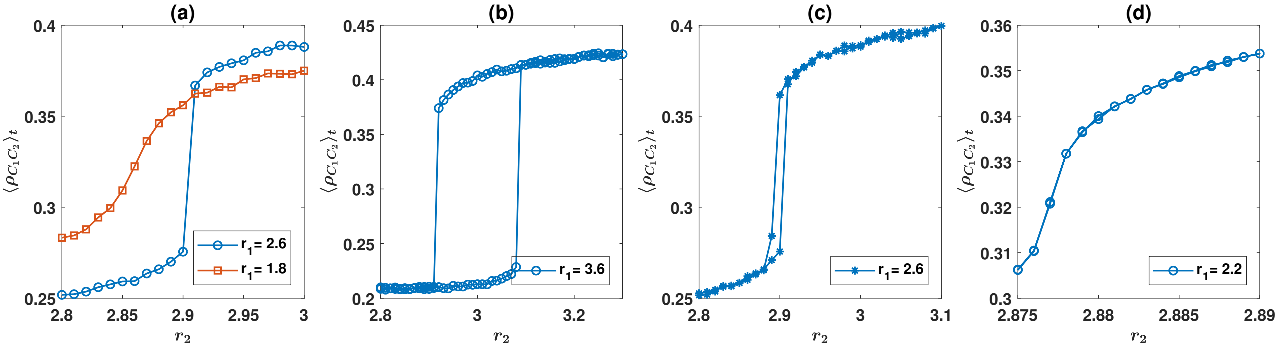

Figure SI.2: Bi-stability of the second scenario model. The time average of $\rho_{C_1C_2}$, as a function of r_2, for two fixed values of r_1 (as indicated in the figures), are plotted. While for small fixed r_1 the transition from the defective to the cooperative periodic orbit is gradual and shows no singularity, this transition becomes singular and happens discontinuously for large r_1. (b) to (d): The hysteresis loops for two different values of r_1. Here, the replicator equations are solved starting with a small value of r_2 below the transition. r_2 is slowly increased up to a large value above the transition and brought slowly back to its initial value. For large r_1 (b and d), depending on the path, the system equilibrates into different states. This shows the system is bistable close to the phase transition. However, for small r_1 (d), the system shows no bistability and the equilibrium state is history independent. Here, $\nu = 10^{-3}$, $g = 10$, $c = 1$, and $\pi_0 = 2$, and the numerical solutions of the replicator equations are used. To derive the hysteresis loops, the replicator dynamics is solved starting with a homogeneous initial condition. After each T time steps, the value of r_2 is changed by a small value (as shown in the figure). The values reported in the figure are averages in the last τ steps, where the system equilibrates. In (b) and (c), $T = 5000$, and $\tau = 3000$, and in (d), $T = 20000$ and $\tau = 5000$. In (a), the replicator dynamics is solved for 5000 time steps, and time averages are taken over the last 2000 steps.

defective PGG can not perform competitively compared to the cooperative PGG, as the vast majority of the individuals who defect in the first round and enter the defective PGG, defect in the second round as well. Consequently, $\rho_{D_1C_2}$ remains small. This PO is called the defective PO, and is plotted by red dotted line in Fig. (SI.1.a) and Fig. (SI.1.c). On the other hand, for larger r_2, in a fixed r_1 (or small r_1 in a fixed r_2) chosen above the transition, cooperation in both defective and cooperative PGGs evolves, and both $\rho_{D_1C_2}$ and $\rho_{C_1C_2}$ take large values. This PO is called the cooperative PO, and is plotted with solid blue line in Fig. (SI.1.a) and Fig. (SI.1.c). The results of a simulation in a population of size $N = 20000$ for the same parameter values is presented in Fig. (SI.1.b) and Fig. (SI.1.d). Comparison reveals a good agreement between the results of a simulations in finite population size and the results of the replicator dynamics. However, in a finite population, population stochasticities can drive fluctuations in the periodic orbits.

In contrast, the first scenario only shows one type of PO, which coincides with the defective PO in the second scenario. This in turn, shows the competitive stability of a reward dilemma to promote cooperation in the face of a potential reward to defection. Two example POs resulting in the first scenario model are presented in Fig. (SI.1.e). Here, $g = 10$, $\nu = 10^{-3}$, $c = 1$, and $\pi_0 = 2$, and the replicator equations are used. To compare, in Fig. (SI.1.f), two POs for the same parameter values, resulted from a simulation in a population of size $N = 20000$ are plotted. Comparison reveals population stochasticities can result in fluctuations in the periodic orbits.

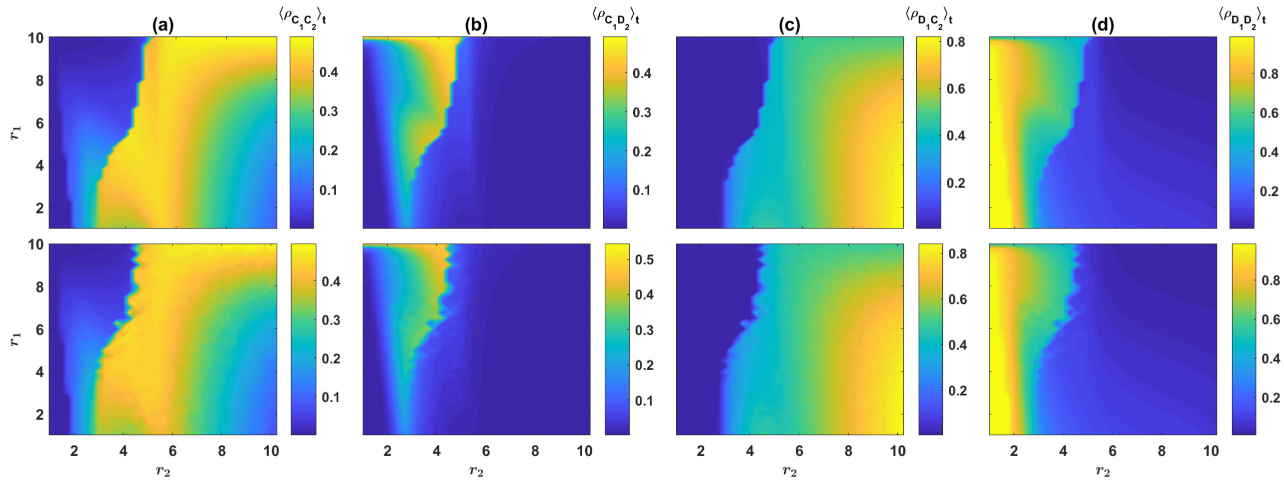

Figure SI.3: The density of different strategies in the second scenario. From (a) to (d), respectively, the time average of $\rho_{C_1C_2}$, $\rho_{C_1D_2}$, $\rho_{D_1C_2}$, and $\rho_{D_1D_2}$ are color plotted in the $r_1 - r_2$ plane. Top panels result form numerical solution of the replicator dynamics, and bottom panels result from a simulation in a population of size $N = 10000$. Here, $g = 10$, $\nu = 10^{-3}$, $c = 1$, and $\pi_0 = 2$. For the analytical solutions, the replicator dynamics is solved for $T = 5000$ time steps and the time average are taken over the last 2000 time steps. The simulation is performed for $T = 4000$ time steps and the time averages are taken over the last 3500 time steps.

SI. 2.2 Phase transition and cross-over between periodic orbits in the second scenario model

We have seen that the second scenario model possesses two different types of periodic orbits, a defective periodic orbit for small r_2, and a cooperative periodic orbit for large r_2. This raises the question that whether a phase transition between these two periodic orbit exist? To answer this question, we need an order parameter, which takes different values for different attractors. We note that the time average of the density of any of the strategies in the population takes different values in the two different POs and can be used as an order parameter. Among these, we choose the density of individuals who cooperate in both games, $\rho_{C_1C_2}$. The time average of this quantity as a function of r_2, for two different values of r_1, is plotted in Fig. (SI.2.a). Here, the replicator dynamics is solved using a homogeneous initial condition in which the density of all the strategies equals. As can be seen, for a fixed large r_1, as r_2 increases, the order parameter changes its value abruptly at a certain value of r_2. This shows the transition from the defective PO to the cooperative PO is discontinuous for large r_1. On the other hand, as can be seen in Fig. (SI.2.a) (for $r_1 = 1.8$), for small r_1, as r_2 increases, there is a crossover from the defective PO to the cooperative PO without passing any singular transition.

The fact that the transition between the two periodic orbits is a discontinuous transition suggests the system can be bistable close to the transition. To see if this is the case, in Fig. (SI.2.b) and Fig. (SI.2.c), we drive the hysteresis loops, for two different values of r_1. Here, we have set $\nu = 10^{-3}$, $g = 10$, $c = 1$, and $\pi_0 = 2$. To drive the hysteresis loops, in Fig. (SI.2.b), we begin solving the replicator dynamics by setting $r_2 = 2.8$, and slowly increase r_2 up to $r_2 = 3.3$, and then decrease it back to the initial value of $r_2 = 2.8$. We plot the time average of $\rho_{C_1C_2}$ as a function of r_2 along the path. Here, for $r_2 = 2.8$, the dynamics settle into the defective PO, where $\langle\rho_{C_1C_2}\rangle_t$ takes a small value. As we gradually increase r_2 the system remains in the defective PO until reaching $r_2 = 3.08$. This can be attested by noting that in this interval the value of $\langle\rho_{C_1C_2}\rangle_t$ changes gradually. Beyond

$r_2 = 3.08$, the defective PO becomes unstable and the system shows an abrupt transition into the cooperative PO. This can be seen in the abrupt change in the value of $\langle \rho_{C_1C_2} \rangle_t$. By increasing r_2 beyond this value, the system remains in the cooperative PO. However, when slowly decreasing r_2 from a large value to the initial value of $r_2 = 2.8$, the system follows a different path. On this path, the system remains in the cooperative PO even below the phase transition value of $r_2 = 3.08$. This shows the system is bistable for some range of r_2 below the phase transition value.

While the system shows hysteresis close to the onset of the cooperative PO for large r_1, the fact that for small r_1 there is a crossover between the two POs suggests the situation is different for small values of r_1. This can be clearly seen to be the case in Fig. (SI.2.d), where $\langle \rho_{C_1C_2} \rangle_t$ is plotted using the same procedure. That is, the replicator dynamics is solved numerically starting with a small value of r_2. Slowly increasing r_2 up to a large value, $\langle \rho_{C_1C_2} \rangle_t$ slowly increases and the system shows a crossover from the defective PO to the cooperative PO. Decreasing r_2 slowly from a large value of r_2 back to its initial value, the system follows the same path. This shows no bistability and hysteresis is at work for small values of r_1.

SI. 2.3 Derivation of the phase diagram and boundaries of bistability

To drive the phase diagram of the second scenario model, we solve the replicator dynamics using a homogeneous initial condition (i.e. $\rho_x = 0.25$, for $x = C_1C_2$, C_1D_2, D_1C_2, and D_1D_2), and for each fixed r_1 identify the value of r_2 where the system shows a phase transition from a fixed point to the defective periodic orbit (blue line in Fig. (2.a) in the main text). Similarly, we identify the value of r_2 where a transition to the cooperative periodic orbit occurs (red line in Fig. (2.a) in the main text). The phase transition line between the defective and cooperative POs in Fig. (2.a) is defined as the region where, starting from a homogeneous initial condition, a transition between these two equilibrium states occur.

To derive the boundaries of bistability in Fig. (2.a), by solving the replicator dynamics for different initial conditions, we check that the transition from the fixed point to the defective periodic orbit shows no bistability and does not depend on the initial condition. Thus, this line coincides with the blue line in Fig. (2.a). Similarly, the transition form the cooperative periodic orbit to the fixed point for large r_2 shows no bistability and occurs in the same parameter values for all the initial conditions. On the other hand, for large enough r_1, the onset of the cooperative periodic orbit when increasing r_2 shows bistability. To derive the lower boundary of the bistability region (that is the value of r_2 below which the cooperative PO is unstable), we solve the replicator dynamics starting with a large value of r_2, above the defective-cooperative PO transition, such that the dynamics settle into the cooperative periodic orbit. We then slowly decrease r_2 in small steps. In each step, as the initial condition, we use the equilibrium state of the dynamics in the last step (i.e. the attractor of the system for a slightly larger r_2). In this way, we are able to find the lower boundary of the bistable region as the largest value of r_2 below which the cooperative PO becomes unstable. We note that this procedure is necessary for deriving the lower boundary of the bistability region, as below the phase transition, although stable, the cooperative PO has a very small basin of attraction. Thus, using different randomly chosen initial conditions, this periodic orbit can remain unobserved.

To derive the upper boundary above which the defective periodic orbit, or the fixed point becomes unstable, similarly to the previous procedure, we start by numerically solving the replicator dynamics for a small enough value of r_2. Then we increase the value of r_2 in small steps, using the equilibrium state of the dynamics for a slightly smaller r_2 as the initial condition for the next step. This allows us to identify the smallest value of r_2 above which the defective periodic orbit or the fixed point becomes unstable as the upper boundary of the bistable region. Interestingly, for small enough r_1, The upper boundary of the bistable region coincides with the phase transition line.

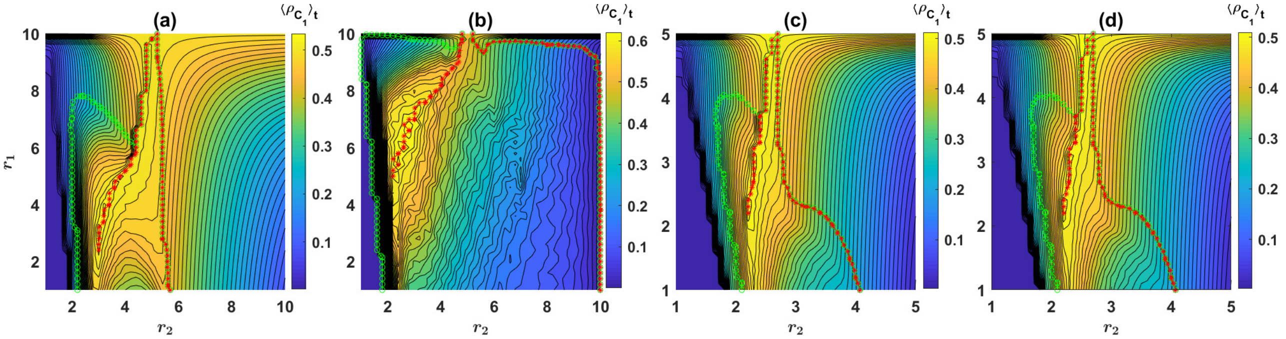

Figure SI.4: Cooperation level in the first round in the second scenario model. Contour plot of $\langle\rho_{C_1}\rangle_t = \langle\rho_{C_1C_2} + \rho_{C_1D_2}\rangle_t$ over $r_1 - r_2$ plane. The lines of phase transitions are marked with green and red markers. For each fixed r_1, $\langle\rho_{C_1}\rangle_t$ is maximized for an intermediate value of r_2, which coincides with the edge of bistability. In (a) $g = 10$, $\nu = 10^{-3}$, in (b) $g = 10$, $\nu = 10^{-5}$, and in (c) and (d) $g = 5$, $\nu = 10^{-3}$. In all the cases the replicator dynamics is solved for $T = 5000$ time steps, and time averages are taken over the last 2000 time steps. In (a) to (c) the initial condition is a homogeneous initial condition in which the initial density of all the strategies equals, and in (d), the $r_1 - r_2$ plane is divided into small cells of linear size 0.1, and a randomly chosen initial condition is used for each cell (as described in the text). In all the cases $c = 1$ and $\pi_0 = 2$.

To derive the phase diagram in the first scenario model, presented in Fig. (1.b), we numerically solve the replicator dynamics to determine different equilibrium states of the system. By solving the replicator dynamics for different initial conditions, we check that the system shows no bistability, and thus, the equilibrium state does not depend on the initial conditions.

SI. 3 The density of different strategies in the second scenario model

The density of different strategies in the second scenario are plotted in Fig. (SI.3). The time average density of strategies, $\rho_{C_1C_2}$, $\rho_{C_1D_2}$, $\rho_{D_1C_2}$, $\rho_{D_1D_2}$ are plotted respectively, in Fig. (SI.3.a), Fig. (SI.3.b), Fig. (SI.3.c), and Fig. (SI.3.d). Top panels result from numerical solutions of the replicator dynamics, and bottom panels result from a simulation in a population of size $N = 10000$. Here, $g = 10$, $\nu = 10^{-3}$, $c = 1$, and $\pi_0 = 2$. As can be seen, the results of replicator dynamics, an exact solution of the model in the infinite population limit, are in good agreement with simulations in finite population size.

For too small values of r_2, the system settles into a defective fixed point with small fraction of cooperators. As r_2 increases, at a certain value of r_2 the cooperative PGG starts to attract individuals. Consequently, the density of the strategies C_1C_2 and C_1D_2 start to increase. This is the region of the phase diagram where the defective PO is stable. In this region, by increasing r_1, $\rho_{C_1C_2}$ decreases, while $\rho_{C_1D_2}$ increases. This shows that while higher enhancement factor of the first game may have a positive effect on the cooperation level in this game, it can have an adverse effect on cooperation in the second game.

By further increasing r_2, the defective PGG starts to attract individuals as well. That is, those who defect in the first round, start to cooperate in the second round as well, and consequently, the defective PGG starts to yield positive reward. At this point, the defective PO becomes unstable, and the dynamics settle into the cooperative PO. In this region, the density of individuals who defect in the second round significantly decreases, and the vast majority of the individuals cooperate in the second round. This is due to the fact that competition between the cooperative and defective PGGs promotes cooperation in the second round. By increasing r_2 in this region, $\rho_{D_1C_2}$ increases while the density

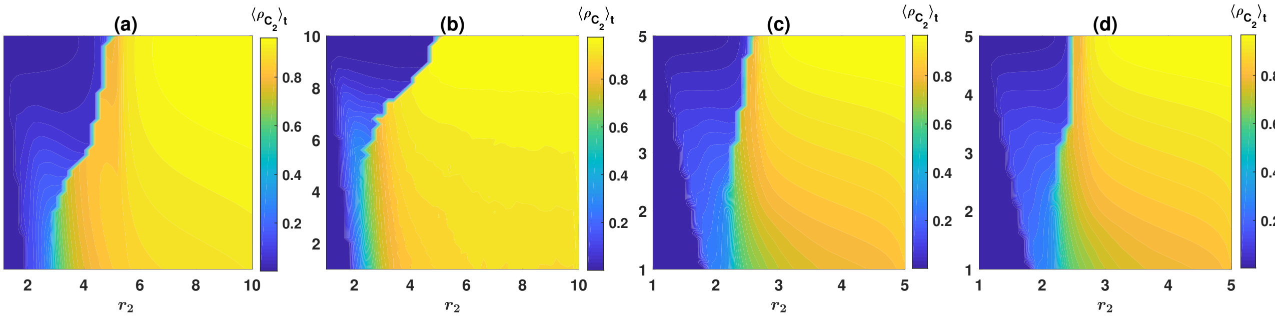

Figure SI.5: Cooperation level in the second round in the second scenario model. Contour plot of $\langle \rho_{C_2} \rangle_t = \langle \rho_{C_1 C_2} + \rho_{D_1 C_2} \rangle_t$ over $r_1 - r_2$ plane. The cooperation level in the second game significantly increases above the transition to the cooperative PO, and monotonically increases by increasing r_2 above this transition. In (a), $g = 10$, $\nu = 10^{-3}$, in (b), $g = 10$, $\nu = 10^{-5}$, and in (c) and (d), $g = 5$, $\nu = 10^{-3}$. In all the cases the replicator dynamics is solved for $T = 5000$ time steps, and time averages are taken over the last 2000 time steps. In (a) to (c) the initial condition is a homogeneous initial condition in which the initial density of all the strategies equals, and in (d), the $r_1 - r_2$ plane is divided into small cells of linear size 0.1, and a randomly chosen initial condition is used for each cell (as described in the text). In all the cases $c = 1$ and $\pi_0 = 2$.

of the other strategies, including $\rho_{C_1 C_2}$ decrease. Thus, when r_2 is too large, the cooperation level in the first game decreases. This in turn results from the same reason that promotes cooperation in the second round: As the competition between cooperative and defective PGGs promote cooperation in the second round, individuals receive a high payoff from the second round irrespective of what they do in the first round and which PGG they enter for the second round. In such circumstances, defection in the first round, which is the rational choice prevails.

Overall, this analysis shows enhancement factors of the game in the two rounds, can have complicated and surprising effect on the cooperation level in these two games. A point to which we will return in the next section.

SI. 4 Dependence of the cooperation level on parameters

In Fig. (SI.4.b) to Fig. (SI.4.d), we plot the contour plot of the time average cooperation level in the first game, $\langle \rho_{C_1} \rangle_t = \langle \rho_{C_1 C_2} + \rho_{C_1 D_2} \rangle_t$, in the $r_1 - r_2$ plane for different parameter values. In Fig. (SI.4.a), $g = 10$ and $\nu = 10^{-3}$, in Fig. (SI.4.b), $g = 10$ and $\nu = 10^{-5}$, and in Fig. (SI.4.c), $g = 5$ and $\nu = 10^{-3}$. In all the cases $\pi_0 = 2$. In these figures, we have also indicated the position of the phase transitions. As can be seen, the same phases appear for different mutation rates and group sizes. However, parameter values can have quantitative effects on the position of the phase transitions and the cooperation evel. Particularly, smaller mutation rates increase the region of the phase space where the dynamics show cyclic behavior.

Interestingly, for a fixed r_1, cooperation level in the first game is maximized for an intermediate value of r_2, such that increasing r_2 beyond this value has a detrimental effect on the level of cooperation in the first game. This is due to the fact that for larger values of r_2 the cooperation level in both cooperative and defective PGGs increase. This decreases the difference in the quality of these two PGGs. While for smaller values of r_2, cooperation level in the defective PGG is much smaller than that in the cooperative PGG. This motivates individuals to cooperate in the first round in order to enter the cooperative PGG. The maximum cooperation level in the first game is achieved exactly on

the transition line between the two periodic orbits, which as shown before, coincides with the edge of bistability.

To see how the initial conditions affect the cooperation level, in Fig. (SI.4.d), we solve the replicator dynamics using different randomly chosen initial conditions. Here, the $r_1 - r_2$ plane is divided into small cells of linear size 0.1, and for each cell a randomly chosen initial condition is used to solve the replicator dynamics. For the initial conditions, we set the initial densities equal to $\rho_{C_1C_2} = a_{C_1C_2}/d$, $\rho_{C_1D_2} = a_{C_1D_2}/d$, $\rho_{D_1C_2} = a_{D_1C_2}/d$, and $\rho_{D_1D_2} = a_{D_1D_2}/d$, where $d = a_{C_1C_2} + a_{C_1D_2} + a_{D_1C_1} + a_{D_1D_2}$, and $a_{C_1C_2}$, $a_{C_1D_2}$, $a_{D_1C_2}$, and $a_{D_1C_2}$, are random numbers drawn uniformly at random in the interval $[0, 1]$. Here, the phase diagram (which is derived using a homogeneous initial condition) is superimposed as well. As can be seen, the initial conditions does not affect the equilibrium state of the system: For all the randomly chosen initial conditions, the dynamics settle in the same equilibrium state as for a homogeneous initial condition. Surprisingly, this is also valid in the bistable region. This shows that, although in the bistable region, different equilibrium states are possible, non-equilibrium states have a very small basin of attraction, such that small deformations in a non-equilibrium state can cause the system to settle into the equilibrium state.

Finally, we study the dependence of the cooperation level in the second game on the parameters. For this purpose, in Fig. (SI.5.a) to Fig. (SI.5.c), we plot the time average cooperation level in the second game, $\langle \rho_{C_2} \rangle_t = \langle \rho_{C_1C_2} + \rho_{D_1C_2} \rangle_t$ in the $r_1 - r_2$ plane. In Fig. (SI.5.a), $g = 10$ and $\nu = 10^{-3}$, in Fig. (SI.5.b), $g = 10$ and $\nu = 10^{-5}$, and in Fig. (SI.5.c), $g = 5$ and $\nu = 10^{-3}$. In all the cases, for small values of r_2, the dynamics settle into, either a fixed point, or the defective periodic orbit. In both cases, cooperation does not evolve in the defective PGG. This keeps the cooperation level small in this region. However, in the region of the phase diagram where the dynamics settle into the cooperative PO, where cooperation in both the defective PGG and the cooperative PGG evolves, cooperative level in the second game significantly increases. In this phase, the cooperation level in the second game monotonically increases by increasing r_2. It is important to notice the situation was completely different in the second scenario model, where only first round cooperators proceed to play a second PGG. As we have seen, in that case, cooperation level in the second game is optimized for an intermediate value of r_2, and increasing $r_2$beyond this optimal value decreases cooperation level in the second game. The different situation in the second scenario model comes from the fact that when first round defectors are allowed to play a second PGG as well, competition between the social (cooperative) and anti-social (defective) PGGs increases, and maintains cooperation in the second round, even when the temptation to free ride is high. This result shows, a potential reward to defection, surprisingly, can further stabilize cooperation.

SI. 5 The evolution of cooperative and defective personalities

As argued in the main text, in our model with the second scenario where both first round cooperators and first round defectors need to choose a strategy in the second round, individuals are more likely to have similar strategies in the two rounds than it can occur by chance. This shows individuals develop consistent personalities in the course of evolution. Here, we show that this result is robust for different parameter regimes.

To see that individuals tend to evolve consistent personalities, we consider two different measures of personality consistency. The first measure is based on the conditional probability that an individual has strategy s_2 in the second round, given that it has strategy s_1 in the first round, $P(s_2|s_1)$. As the first measure of the consistency of individual's strategies in the two rounds, we define the personality consistency measure, γ, as $\gamma = [P(C_2|C_1) + P(D_2|D_1) - P(D_2|C_1) - P(C_2|D_1)]/2$. As a second measure, we consider the connected correlation function between the strategies of the individuals in

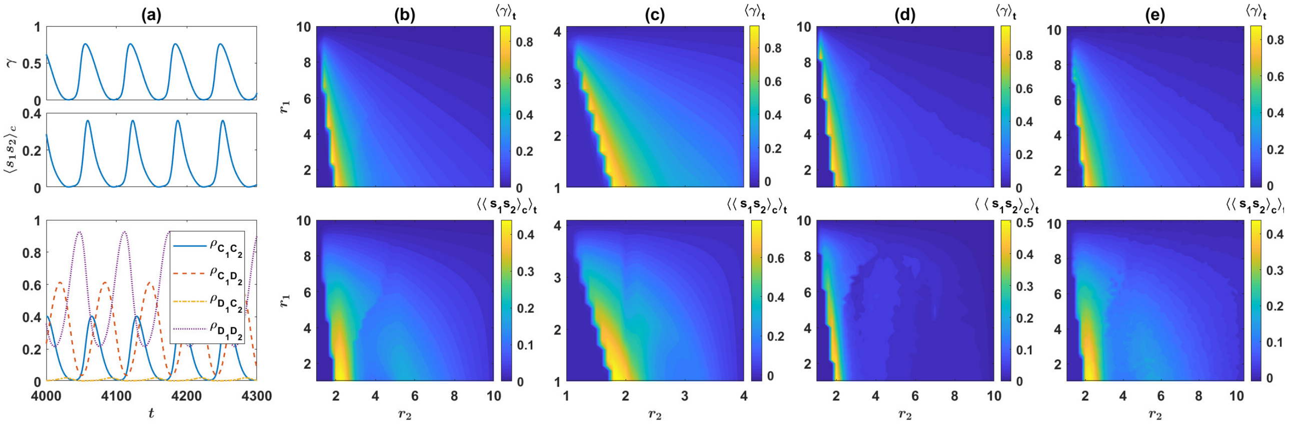

Figure SI.6: Evolution of consistent personalities. (a) The measure of consistency in personality, defined as $\gamma = [P(C_2|C_1) + P(D_2|D_1) - P(D_2|C_1) - P(C_2|D_1)]/2$ (top), and the connected correlation function between strategies of individuals in the two rounds, $\langle s_1 s_2 \rangle_c = \langle s_1 s_2 \rangle - \langle s_1 \rangle \langle s_2 \rangle$ (middle), as a function of time in the stationary state. The density of different strategies is plotted as well (down). Both measures remain always non-negative. This shows individuals are more likely to have the same strategies in the two rounds, and consistent cooperative or defective personalities evolve. (b), (c), (d) and (e): Contour plot of the time average of γ (top), and the time average of $\langle s_1 s_2 \rangle_c$ (bottom), in the $r_1 - r_2$ plane, for different parameter values. In (a) to (d), the replicator dynamics are numerically solved for $T = 5000$ time steps, and time averages are taken over the last 2000 time steps. In (e), a simulation on a population of size $N = 5000$ for $T = 3000$ time steps is performed, and the time averages are taken after discarding the first 500 time steps. In (a), (b), and (e) $g = 10$ and $\nu = 10^{-3}$, in (c) $g = 4$ and $\nu = 10^{-3}$, and in (d) $g = 10$ and $\nu = 10^{-5}$. In (a) $r_1 = 2.8$ and $r_2 = 4.8$. In all the cases $c = 1$ and $\pi_0 = 2$.

the two rounds $\langle s_1 s_2 \rangle_c = \langle s_1 s_2 \rangle - \langle s_1 \rangle \langle s_2 \rangle$. Here, $\langle . \rangle$ denotes an average over the population. To calculate this, we assign -1 to the strategy D, and $+1$ to the strategy C.

Both measures always lie between -1 and $+1$. A positive value shows that individuals are more likely to have consistent strategies in the two rounds, and a negative value indicates that individuals are more likely to have opposite strategies in the two rounds. In Fig. (SI.6.a), we plot γ (top panel) and $\langle s_1 s_2 \rangle_c$ (middle panel) in the stationary state, as a function of time. Here, the replicator dynamics is used, and $g = 10$, $\nu = 10^{-3}$, $c = 1$, $\pi_0 = 2$, $r_1 = 2.8$ and $r_2 = 4.8$. As can be seen, both γ and $\langle s_1 s_2 \rangle_c$ follow a cyclic behavior. Importantly, When the density of consistent C_1C_2 and D_1D_2 strategies are high, γ and $\langle s_1 s_2 \rangle_c$ take a large value and they drop as inconsistent C_1D_2 and D_1C_2 strategies reach a high fraction. Furthermore, γ and $\langle s_1 s_2 \rangle_c$ always remain non-negative. This shows strategies always remain consistent. To see how the consistency of the strategies change in the whole parameter regime, in Fig. (SI.6.b) to Fig. (SI.6.d), we plot the time average of γ (top panels) and $\langle s_1 s_2 \rangle_c$ (bottom panels), over $r_1 - r_2$ plane. In Fig. (SI.6.a), $g = 10$ and $\nu = 10^{-3}$, in Fig. (SI.6.c) $g = 4$ and $\nu = 10^{-3}$, and in Fig. (SI.6.d), $g = 10$ and $\nu = 10^{-5}$. In all the case $c = 1$ and $\pi_0 = 2$. Here, the replicator-mutation equations are solved for $T = 5000$ time steps, and time averages are taken over the last 2000 time steps. As can be seen, in all the cases both measures remain non-negative. For small r_2, such that the system remains in the defective fixed point and D_1D_2 strategies prevail, both measures take a small value close to zero. This shows that in this regime, although strategies seem to be consistent, however, there is no correlation between the strategies in the two rounds, and thus our measures predict no personality consistency. As the frequency of non-defective strategies start to increase, both measures start to become large, which shows the existent strategies start to become consistent.

To see that the results agree with simulations in finite populations, in Fig. (SI.6.e) a simulation on a population of size $N = 5000$ is used. Here $g = 10$, $\nu = 10^{-3}$, $c = 1$ and $\pi_0 = 2$. This corresponds to the parameter values used in Fig. (SI.6.b). As can be seen, our results are valid in finite populations as well. In addition, a good agreement can be seen between analytical predictions, valid in the infinite population limit, and the simulation results in finite population size.
